# Stromal Hedgehog signalling is downregulated in colon cancer and its restoration restrains tumour growth

**DOI:** 10.1038/ncomms12321

**Published:** 2016-08-05

**Authors:** Marco Gerling, Nikè V. J. A. Büller, Leonard M. Kirn, Simon Joost, Oliver Frings, Benjamin Englert, Åsa Bergström, Raoul V. Kuiper, Leander Blaas, Mattheus C. B. Wielenga, Sven Almer, Anja A. Kühl, Erik Fredlund, Gijs R. van den Brink, Rune Toftgård

**Affiliations:** 1 Center for Innovative Medicine, Department of Biosciences and Nutrition, Karolinska Institutet, NOVUM, Hälsovägen 7, 14183 Huddinge, Sweden; 2 Tytgat Institute for Liver and Intestinal Research and Department of Gastroenterology and Hepatology, Academic Medical Center, Meibergdreef 69-71, AZ1105 Amsterdam, The Netherlands; 3 Department of Medicine I for Gastroenterology, Infectious Diseases and Rheumatology, Charité, Campus Benjamin Franklin, Hindenburgdamm 30, 12200 Berlin, Germany; 4 Department of Oncology-Pathology, Science for Life Laboratory, Karolinska Institutet, 17176 Stockholm, Sweden; 5 Core Facility for Morphologic Phenotype Analysis, Clinical Research Center, Karolinska Institutet, Hälsovägen 7-9, 14183 Huddinge, Sweden; 6 Department of Medicine, Solna, Karolinska Institutet, 17176 Stockholm, Sweden; 7 Center for Digestive Diseases, Karolinska University Hospital, 17176 Stockholm, Sweden

## Abstract

A role for Hedgehog (Hh) signalling in the development of colorectal cancer (CRC) has been proposed. In CRC and other solid tumours, Hh ligands are upregulated; however, a specific Hh antagonist provided no benefit in a clinical trial. Here we use Hh reporter mice to show that downstream Hh activity is unexpectedly diminished in a mouse model of colitis-associated colon cancer, and that downstream Hh signalling is restricted to the stroma. Functionally, stroma-specific Hh activation in mice markedly reduces the tumour load and blocks progression of advanced neoplasms, partly via the modulation of BMP signalling and restriction of the colonic stem cell signature. By contrast, attenuated Hh signalling accelerates colonic tumourigenesis. In human CRC, downstream Hh activity is similarly reduced and canonical Hh signalling remains predominantly paracrine. Our results suggest that diminished downstream Hh signalling enhances CRC development, and that stromal Hh activation can act as a colonic tumour suppressor.

## Abstract (web summary)

The Hedgehog signalling pathway can drive tumorigenesis. Here, the authors show that in a colitis-associated colon cancer model downstream Hedgehog signalling is restricted to the stroma and its over-activation can inhibit tumorigenesis, associated with activation of BMP signaling.

## Graphical abstract

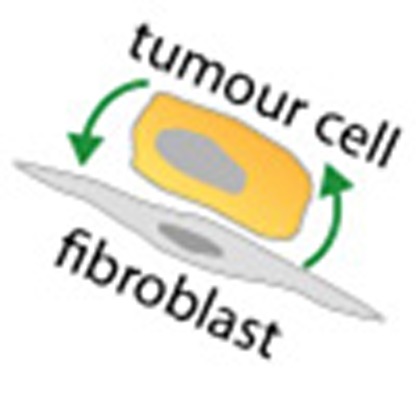

The Hedgehog (Hh) signalling pathway is one of the major regulators of embryonic development. Later in life, it can drive tumorigenesis: mutations that lead to cell-autonomous Hh pathway activation cause nearly all basal cell carcinomas, as well as subsets of medulloblastomas and rhabdomyosarcomas^1^. By contrast, the majority of solid tumours, including colorectal cancer (CRC), rarely carry mutations in Hh genes^2^. Instead, in CRC, Hh ligand expression is upregulated and conflicting data suggest either a paracrine role for Hh ligands in shaping a tumour-supportive microenvironment^3^ or autocrine pathway activation that promotes metastasis^4^.

Under homeostatic conditions, the main intestinal ligand, Indian hedgehog (Ihh), is secreted by differentiated enterocytes, whereas downstream signalling is activated exclusively in the stroma^5^. In the ‘canonical' Hh signalling cascade, binding of the ligand to the inhibitory receptor Patched 1 (Ptch1) leads to derepression of the activating receptor Smoothened (Smo), thereby initiating a signalling cascade that culminates in the stromal activation of the Glioma-associated oncogene (Gli) proteins, Gli1, Gli2 and Gli3 (ref. 5). *Gli1* expression is considered the most reliable indicator of downstream pathway activity, whereas Hh interacting protein (*Hhip*), *Ptch1* and its homologue *Ptch2* are further common downstream targets^6^.

The SMO antagonist, vismodegib, has recently been approved for the treatment of basal cell carcinomas^7^. Motivated by the upregulation of Hh ligands in CRC, a clinical trial with vismodegib added to first-line therapy in metastatic CRC was recently completed, but yielded a negative result^8^. A trial with vismodegib in pancreatic cancer, in which Hh ligands are similarly overexpressed, had an equally discouraging outcome^9^, whereas a further pancreatic cancer study using another SMO antagonist was halted due to an inferior outcome in the inhibitor-treated group^10^. Collectively, these clinical data challenge the paradigm of a tumour-promoting stroma shaped by Hh signalling and contest a putative oncogenic role for cancer cell-autonomous Hh activation in these tumour types.

Functionally, the stromal response to the Hh ligand is part of a paracrine loop that controls differentiation of the intestinal epithelium^11^. Diminished Hh signalling evokes an expansion of the intestinal stem cell compartment and leads to impaired enterocyte differentiation, as well as activation of Wnt signalling, the central oncogenic driver pathway in CRC^11^,^12^,^13^,^14^.

Given this discrepancy between data suggesting a tumour-promoting role for Hh in CRC on one hand and its pro-differentiating role under homeostatic conditions, together with negative results from clinical trials, on the other hand, we sought to get a more precise picture of the role played by Hh in colorectal tumourigenesis. Using Hh reporter mice, we provide evidence that downstream Hh signalling activity is reduced in murine colon tumours. Functionally, diminished Hh signalling promotes colitis-associated colonic tumourigenesis in mice, whereas stroma-specific Hh activation markedly curtails tumour development. Similarly, human CRCs harbour diminished expression of Hh downstream targets despite upregulated expression of the ligand, Sonic Hh (*SHH*). Together, our data suggest that stromal activation of Hh signalling can function as a suppressor of colorectal carcinogenesis.

## Results

### Reduced stromal Hh signalling in murine colonic tumours

**Figure 1:**
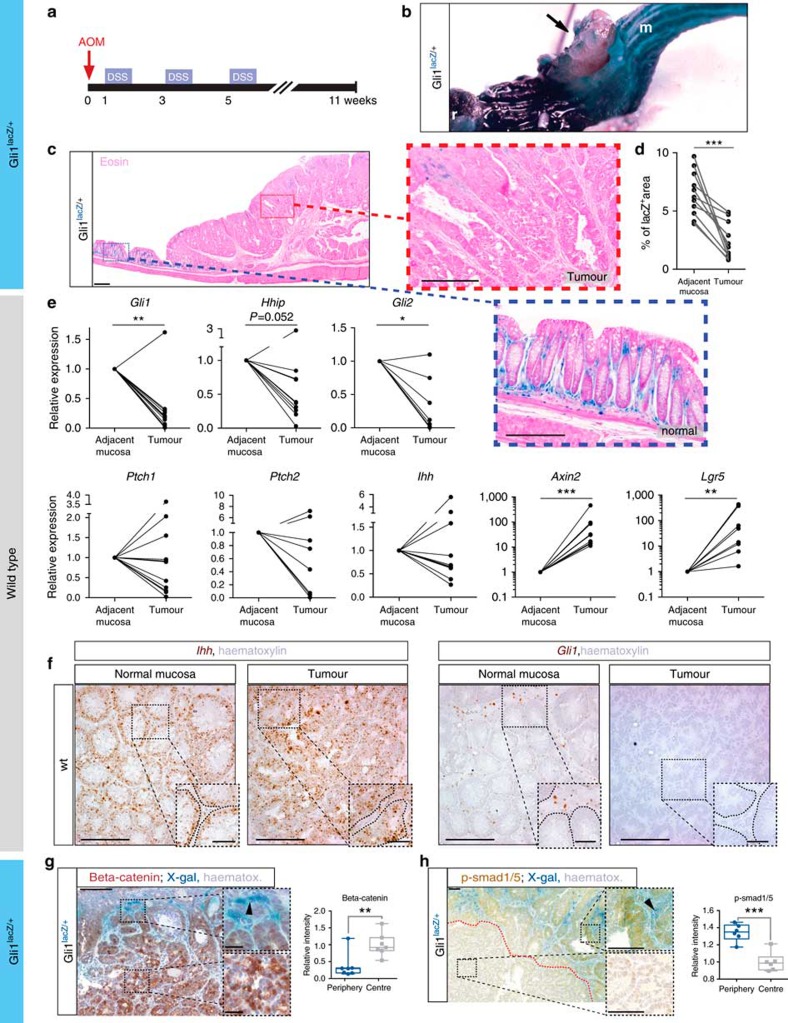
Reduced stromal Hh activity in AOM/DSS-induced colon tumours. (**a**) Schematic of the AOM/DSS protocol. (**b**) Representative example of an X-gal-stained tumour in a *Gli1*^*lacZ/+*^ mouse (from >20 tumours in 11 mice). Arrow indicates the tumour; r, rectum; m, normal mucosa. (**c**) Microscopic appearance of an AOM/DSS-induced tumour in a *Gli1*^*lacZ/+*^ mouse after X-gal staining (representative of *n*=11 tumours from *n*=9 mice). Magnifications of normal mucosa: blue box and neoplastic tissue: red box; scale bars, 100/50 μm. (**d**) Quantification of X-gal-positive area in tumour and adjacent mucosa (*P*=0.0002; paired *t*-test). (**e**) Real-time quantitative PCR (RT-qPCR) for the indicated transcripts from tumours and matched normal mucosa, statistics from paired *t*-tests based on the ΔCT values with multiple test correction (fdr, Benjamini and Hochberg): *P*=0.003 (*Gli1*), *P*=0.052 (*Hhip*), *P*=0.0232 (*Gli2*), *P*=0.199 (*Ptch1*), *P*=0.314 (*Ptch2*), *P*=0.771 (*Ihh*), *P*<0.0001 (*Axin2*) and *P*=0.0016 (*Lgr5*); *n*=11 tumours and adjacent mucosa from 11 wt mice; at least 8 matched pairs analysed for each transcript. (**f**) RNA ISH for *Ihh* and *Gli1* on consecutive sections of the same tumour/mucosa sample; scale bars, 50/10 μm; dashed lines in magnified sections denote epithelial compartment. (**g**) IHC of β-catenin in an X-gal-stained *Gli1*^*lacZ/+*^ tumour indicating mutual exclusivity of epithelial Wnt activation and stromal Gli1; scale bars, 500/50 μm. The graph compares the relative staining intensity of the DAB chromophore at the tumour periphery and centre (*n*=7 tumours, *P*=0.006; box plots; whiskers represent minima and maxima). Arrowhead points to area of positive X-gal staining. (**h**) IHC of p-Smads1/5 in a tumour from a *Gli1*^*lacZ/+*^ mouse. Scale bars, 100 μm. Quantification of DAB intensity shows an increase at the periphery, in parallel to stromal Gli1 (*n*=6 tumours, *P*=0.0002; unpaired *t*-tests in **f** and **g**); data presented as box-and-whisker plots, whiskers represent minima and maxima. Arrowheads point to cells with positive X-gal staining indicating Gli1 expression. *P<0.05, **P<0.01 and ***P<0.001.

To induce colonic tumours in mice, we employed a chemical model based on injection of the mutagenic agent azoxymethane (AOM), followed by repeated oral treatment with dextran sodium sulphate (DSS) to induce epithelial damage and subsequent colitis (Fig. 1a)^15^. Tumours in this model recapitulate central aspects of human CRCs as they consistently exhibit mutations in the genes for adenomatous polyposis coli (*Apc*) or catenin β1 (*Ctnnb1*)^16^, and, less frequently, in *Kras*^17^. We consider this model particularly well suited to our purposes, as it can be combined readily with *Cre-LoxP* models to modify epithelial and mesenchymal Hh signalling, and because tumours arise specifically in the colon but not in the small intestine.

To assess downstream Hh signalling activity, we first subjected *Gli1* reporter mice harbouring a β-galactosidase knock-in to *Gli1* (*Gli1*^*lacZ/+*^ mice^18^), to AOM/DSS treatment, and then visualized Gli1 expression with whole-mount X-gal staining. Although non-malignant mucosa stained strongly, X-gal staining was weak to absent in AOM/DSS-induced tumours (Fig. 1b). Histological analysis revealed Gli1 expression exclusively in the stroma and reduced X-gal staining in the tumours (Fig. 1c,d).

Real-time quantitative PCR confirmed reduced *Gli1* expression in tumours from wild-type (wt) mice, with a congruent reduction of the Hh targets, *Hhip* and *Gli2* (Fig. 1e). Remarkably, expression of the receptor, *Ptch1*, and its homologue, *Ptch2*, as well as the main intestinal ligand, *Ihh*, was dissociated from attenuated downstream Hh signalling (Fig. 1e), whereas all tumours demonstrated increased Wnt activity, as expression of the Wnt targets, *Axin2* and Leucine-rich repeat-containing G-protein coupled receptor 5 (*Lgr5*) was upregulated (Fig. 1e). RNA *in situ* hybridization (ISH) confirmed that downregulation of stromal *Gli1* can occur despite high *Ihh* expression in the adjacent tumour cells (Fig. 1f).

Given that Hh downstream signalling in the intestine is exclusively stromal^5^, the reduced expression of downstream Hh targets could be the result of a reduction in the number of stromal cells. To explore this possibility, we used immunofluorescence (IF) to stain for the intermediate filaments desmin, vimentin and α-smooth muscle actin simultaneously. This marker combination covers the majority of normal and cancer-associated non-inflammatory stromal cells, for which no specific single marker exists^19^. However, we saw no differences in the amount of stromal cells between normal mucosa and adjacent tumours, indicating that stromal cell loss is not the reason for diminished expression of Hh downstream targets (Supplementary Fig. 1a–c).

Immunohistochemistry (IHC) of β-catenin in tumours from *Gli1*^*lacZ/+*^ mice revealed its nuclear translocation in areas of low stromal Gli1 expression (Fig. 1g), substantiating a model in which the stromal downstream Hh signal is diminished in areas of high epithelial Wnt activity. In line with this, we found that the expression of p-Smads1/5—markers of epithelial differentiation driven by bone morphogenetic protein (BMP) signalling that are highly expressed in differentiated enterocytes^20^—correlated spatially with stromal Gli1 (Fig. 1h).

Hence, colonic tumour development in the AOM/DSS model is unexpectedly associated with a diminution in the stromal Hh response. To explore Hh expression in a different colon cancer model, we probed RNA-sequencing data from tumours in which Apc activity is regulated by a doxycycline-dependent short hairpin RNA (shRNA) (Gene Expression Omnibus (GEO) data set GSE67186 and ref. 21). We found that these non-inflammatory colon tumours recapitulated the expression pattern of the AOM/DSS model, in that expression of the downstream Hh targets, *Gli1*, *Gli2*, as well as *Gli3*, was reduced significantly and was dissociated from expression of the ligands, *Ihh* (unchanged) and *Shh* (increased), as well as the receptor *Ptch1* (increased) (Supplementary Fig. 2a–c).

### Increased tumour burden upon diminished Hh signalling

**Figure 2:**
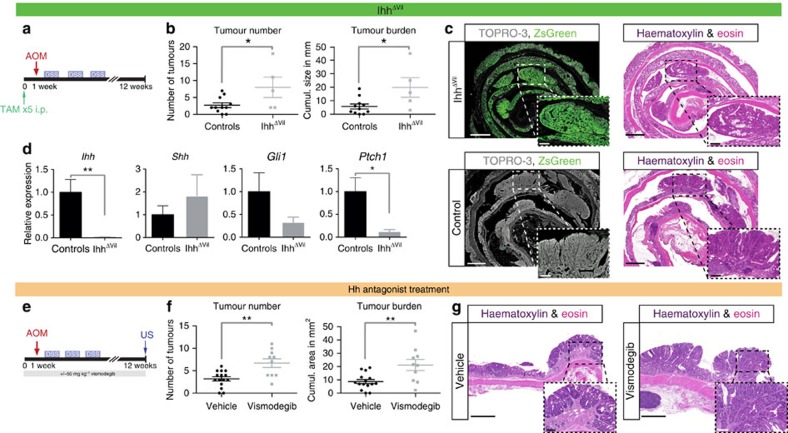
Reduced Hh signalling increases tumour burden in the AOM/DSS model. (**a**) Schematic of Tam and AOM/DSS treatment of *Ihh*^*ΔVil*^ mice. (**b**) Tumour numbers and tumour burden for *Ihh*^*ΔVil*^ mice (*n*=13) and controls (*n*=11 littermates that lacked either the *VillinCreER* allele or floxed *Ihh* alleles or both): 5/13 *Ihh*^*ΔVil*^ mice and 11/11 controls reached the endpoint; *P*=0.0321 (tumour numbers), *P*=0.0215 (tumour burden; both *t*-tests). (**c**) Representative confocal image of ZsGreen reporter fluorescence in an *Ihh*^*ΔVil*^ mouse (upper left panel). Persistent reporter expression indicates enduring loss of *Ihh* in the tumour. Haematoxylin and eosin (H&E) staining of consecutive section (upper right panel). Littermate wt controls are shown in the lower panels. Dashed boxes show magnified areas. Nuclear counterstain: TOPRO-3. Scale bars, 1 mm/250 μm. Representative image of *n*=2 *Ihh*^*ΔVil*^ mice with *n*=8 tumours, in which all tumours expressed ZsGreen, whereas no zsGreen expression was detected in *n*=3 control mice. (**d**) Real-time quantitative PCR (RT-qPCR) for the indicated transcripts in *Ihh*^*ΔVil*^ mice (*n*=5) and controls (*n*=5); *P*=0.003 (*Ihh*), *P*=0.887 (*Shh*), *P*=0.311 (*Gli1*), *P*=0.0142 (*Ptch1*; all *t*-tests, *P*-values after multiple test correction (fdr)). Bars represent mean and whiskers represent s.e.m. (**e**) Schematic of tumour induction, vismodegib treatment and tumour quantification with US; wt C57BL/6 mice received either 25 mg kg^−1^ vismodegib or vehicle (DMSO) twice daily, 6 days per week. (**f**) Tumour numbers and tumour burden for mice treated with vehicle (*n*=16) or vismodegib (*n*=15): 15/16 DMSO-treated and 10/15 vismodegib-treated mice reached the endpoint; *P*=0.0014 (tumour numbers), *P*=0.0036 (tumour burden; both *t*-tests). (**g**) Representative histology of tumours from the indicated treatment groups (H&E); scale bars, 1 mm/200 μm. *P<0.05, **P<0.01 and ***P<0.001.

In the murine colon, *Ihh* messenger RNA expression exceeds that of *Shh* by several orders of magnitude (Supplementary Fig. 2a and refs 22, 23), and an *Shh* conditional knockout model revealed a limited phenotype strictly confined to the terminal ileum^24^. To assess whether reduced Hh signalling can promote malignant transformation in the colon, we used a genetic model in which the main intestinal ligand, *Ihh*, can be knocked out by Tamoxifen (Tam) administration to *VillinCreER;Ihh*^*flox/flox*^*;R26-LSL-ZsGreen* mice (hereafter *Ihh*^*ΔVil*^), simultaneously activating expression of a conditional fluorescent reporter. Although prolonged loss of *Ihh* in intestinal epithelial cells leads to intestinal stem cell accumulation, loss of differentiated cells and activation of the Wnt pathway, it does not, by itself, result in adenoma formation^12^. However, when we exposed *Ihh*^*ΔVil*^ mice to AOM/DSS (Fig. 2a), we found a significant increase in the tumour burden compared with controls (Fig. 2b). The ZsGreen reporter (indicating successful *Cre*-mediated recombination) persisted throughout normal and tumourous epithelium, demonstrating a permanent loss of *Ihh* and strongly suggesting that tumour development is not dependent on *Ihh* expression (Fig. 2c). Indeed, *Ihh* mRNA was barely detectable in *Ihh*^*ΔVil*^ mice, *Shh* did not increase significantly and stromal downstream Hh targets were reduced (Fig. 2d and Supplementary Fig. 3a,b). It is worth noting that *Ihh*^*ΔViI*^ mice lost more weight than controls after DSS treatment and had a higher mortality, which may imply that enhanced inflammatory activity contributes, at least partly, to the increased tumour frequency in this model (Supplementary Fig. 3c,d).

In a subsequent experiment, we used vismodegib to antagonize canonical Hh signalling at the level of the receptor Smo (Fig. 2e). Twice daily administrations of vismodegib (25 mg per kg body weight) led to a significant reduction in downstream Hh target gene expression (Supplementary Fig. 3e). We treated C57BL/6 wt mice with either vismodegib or vehicle and employed high-frequency ultrasound (US) with intraluminal contrast to assess polypoid colonic lesions at high resolution (Supplementary Movies 1 and 2 and ref. 25). We found that inhibition of canonical Hh signalling with vismodegib had an effect similar to the *Ihh* knockout, in that it led to more frequent and larger tumours (Fig. 2f,g). However, we observed no significant differences in terms of weight loss or mortality due to colitis (Supplementary Fig. 3f,g).

Taken together, the data demonstrated that canonical downstream Hh signalling is attenuated in murine colorectal carcinogenesis, and that Hh inhibition can further accelerate tumour development.

### Stroma-specific activation of Hh signalling

**Figure 3:**
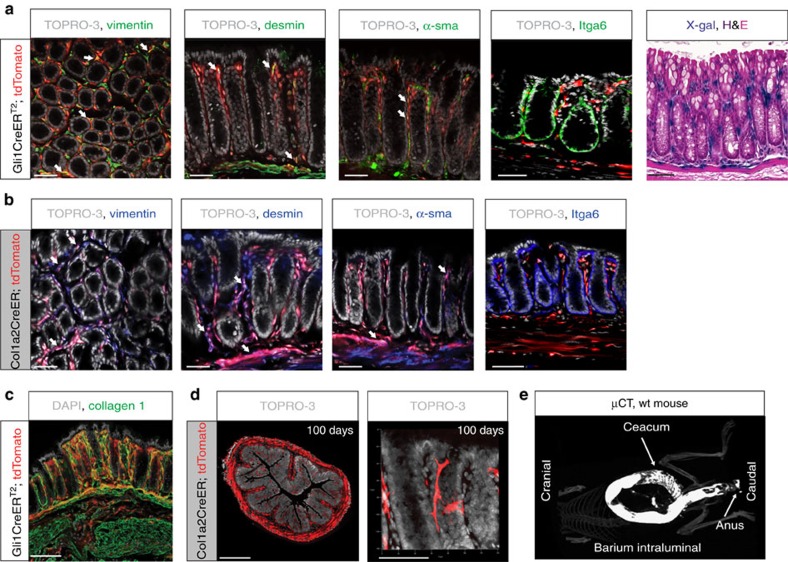
Stroma-specific Cre-recombination in the murine colon. Initial tracing (7 days after Tam) and IF staining for the stromal proteins vimentin, desmin, α-sma and the epithelial protein, integrin α-6, with TOPRO-3 as a nuclear counterstain: (**a**) *Gli1CreERT2;R26-LSL-tdTomato* mouse, representative images from *n*=3 mice. Comparison with the *Gli1*^*lacZ/+*^ model in the right panel; scale bars, 50 μm. (**b**) *Col1a2CreER;R26-LSL-tdTomato* mouse with representative images from *n*>3 mice for each staining. Scale bars, 50 μm. (**c**) 3D rendering of a 25 μm-thick colon section from a *Gli1CreERT2;R26-LSL-tdTomato* mouse 7 days after Tam treatment, stained for collagen-1 (Col1). Overlap of Gli1 and Col1 in yellow. Nuclear counterstain: 4,6-diamidino-2-phenylindole (DAPI). Scale bar, 100 μm. (**d**) Tomato^+^ cells under the control of *Col1a2CreER* 100 days after Tam injection; scale bars, 500/50 μm. (**e**) Barium contrast enema illustrating colonic specificity of intraluminal 4OH-Tam instillations via the rectum (see also Supplementary Fig. 5).

To visualize Hh target cells in a near-native setting, we crossed *Gli1CreER*^*T2*^ mice to *Rosa26-LSL-tdTomato* reporter mice, yielding inducible *Gli1CreER*^*T2*^*;Rosa26-LSL-tdTomato* mice. Seven days after administration of Tam, we found *tdTomato*^*+*^ cells exclusively residing in the stroma, including a mixed population of cells expressing the cytoskeletal proteins desmin, vimentin and α-sma (Fig. 3a). Gli1 expression remained exclusively stromal after DSS-induced epithelial damage, as indicated by analysis of both the *Gli1*^*lacZ/+*^ and *Gli1CreERT2;tdTomato* reporter mice (Supplementary Fig. 4a–d), indicating that downstream Hh signalling is not activated ectopically in damaged epithelium.

In *Gli1CreER*^*T2*^ mice, *Cre* is knocked in to the endogenous *Gli1* locus^18^; thus, the loss of one functional *Gli1* allele alters downstream Hh signalling. To circumvent this problem in further experiments aimed at activating Hh signalling specifically in stromal cells, we used mice expressing *Cre* from a transgenic collagen, type I, α2 (*Col1a2*) promoter^26^. Analysis of Tam-induced *Col1a2CreER;R26-LSL-tdTomato* reporter mice confirmed recombination in an exclusively stromal cell population (Fig. 3b) and collagen 1 protein expression overlapped with tdTomato expression in *Gli1CreER*^*T2*^*;Rosa26-LSL-tdTomato* mice (Fig. 3c). We found that *Col1a2CreER* targets a persistent cell population as indicated by abundant tdTomato^+^ fibroblasts 100 days after recombination (Fig. 3d). As the *Col1a2* transgene is expressed in a wide range of organs^26^, we administered the active metabolite 4OH-Tam intraluminally, to minimize systemic effects in subsequent experiments, where we employed *Col1a2CreER*-expressing mice to activate Hh signalling specifically in the stroma (Fig. 3e and Supplementary Fig. 5).

### Hh activation antagonizes colonic tumour development

**Figure 4:**
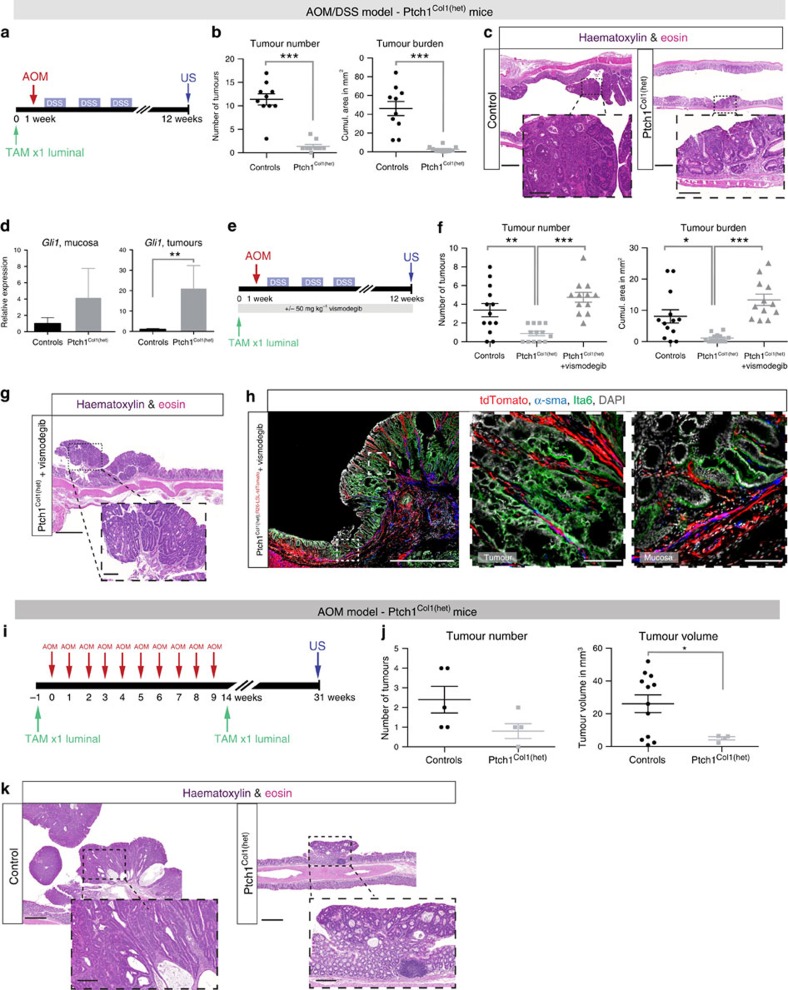
Stromal Hh activation attenuates colonic tumour development. (**a**) Experiment schematic; controls comprise littermates lacking either the floxed *Ptch1* allele or the *Cre* recombinase, or both. (**b**) Tumour numbers and cumulative tumour sizes in controls (*n*=12 of which 10 reached the endpoint) and *Ptch1*^*Col1(het)*^ mice (*n*=12 of which 10 reached the endpoint). *P*<0.0001 (for both tumour number and burden, *t*-tests). (**c**) Histology (haematoxylin and eosin (H&E)) of representative tumours from each group presented in **b**); scale bars, 1 mm/200 μm. (**d**) *Gli1* mRNA expression in normal mucosa (*n*=8 controls and *n*=6 *Ptch1*^*Col1(het)*^ mice; *P*=0.235) and tumours (*n*=10 tumours from controls and *n*=9 tumours from *Ptch1*^*Col1(het)*^ mice; *P*=0.002); *t*-test statistics on ΔCT values. Bars represent means and whiskers represent s.e.m. (**e**) Schematic of vismodegib administration (25 mg kg^−1^ body weight twice daily, 6 days per week), Tam administration and tumour induction. (**f**) Tumour numbers and cumulative sizes in controls (littermates as defined in **b** that received Tam and twice daily injections of dimethyl sulfoxide (DMSO); *n*=17 of which 13 reached the endpoint), *Ptch1*^*Col1(het)*^ mice (*n*=13 of which all reached the endpoint) and *Ptch1*^*Col1(het)*^ mice (treated with vismodegib twice daily, 6 days per week, *n*=19 of which 12 reached the endpoint). *P*-values from one-way analysis of variance with Tukey's multiple comparison test are indicated. (**g**) Representative histology (H&E staining) of a *Ptch1*^*Col1(het)*^ mouse treated with vismodegib. (**h**) Confocal image of a *Ptch1*^*Col1(het)*^ mouse with an inducible *R26-LSL-tdTomato* reporter, treated with vismodegib. TdTomato^+^ cells are exclusively stromal in tumour (middle panel representing magnification of boxed area in the left panel) and normal tissue (right panel magnification); stromal marker: α-smooth muscle actin (α-sma) (blue); epithelial marker: Itα6 (green); nuclear stain: 4,6-diamidino-2-phenylindole (DAPI). Scale bars, 1 mm/100 μm. Representative of *n*=4 tumours in *n*=2 mice. (**i**) Schematic of AOM injections and Tam administration (repeated as Col1a2^+^ progeny were known to persist at least for up to 100 days; Fig. 3d). (**j**) Tumour numbers and volumes for *Ptch1*^*Col1(het)*^ mice and controls (*n*=6 littermates as defined in **b**, of which five (controls) and four (AOM) reached the endpoint; *P*=0.143 and *P*=0.045, respectively, *t*-tests). (**k**) Representative histology of the AOM-induced tumours for the indicated genotypes. Scale bars, 1 mm/200 μm. Whiskers represent s.e.m.; *P<0.05, **P<0.01 and ***P<0.001.

To activate Hh signalling in *Col1a2*^*+*^ cells, we crossed *Col1a2CreER* mice to *Ptch1neo[Δ]Ex2[Δ]* mice with LoxP-sites flanking exon 2 of *Ptch1* (ref. 27), creating *Col1a2CreER;Ptch1*^*flox/+*^ mice (hereafter *Ptch1*^*Col1(het)*^mice). In this model, Tam treatment leads to the knockout of one *Ptch1* allele specifically in *Col1a2*-expressing stromal cells. Challenged with AOM/DSS (Fig. 4a), *Ptch1*^*Col1(het)*^ mice developed significantly fewer and smaller tumours than controls, revealing a protective effect of stromal Hh activation on colorectal carcinogenesis (Fig. 4b,c). Following the loss of one *Ptch1* allele, *Gli1* expression increased significantly in tumour tissue, whereas the effect in normal mucosa was less pronounced, probably reflecting background Hh activity in homeostasis (Fig. 4d). In line with this, we found no phenotypical differences between wt littermates and *Ptch1*^*Col1(het)*^ mice during a 6-month follow-up after recombination (data not shown). Moreover, we observed no differences between *Ptch1*^*Col1(het)*^ mice and control animals with respect to their sensitivity to DSS treatment or mucosal immune cell composition at the endpoint of the AOM/DSS protocol (Supplementary Fig. 6a,b). In tumours from *Ptch1*^*Col1(het)*^ mice, *Gli1* transcripts were enriched in the stromal compartment as expected (Supplementary Fig. 6c,d), yet the amount of stroma did not differ significantly between tumours from control animals and those from *Ptch1*^*Col1(het)*^ mice, as assessed by IF (Supplementary Fig. 6e). Importantly, treatment of *Ptch1*^*Col1(het)*^ mice with vismodegib (Fig. 4e) overcame the protective effect of Hh activation, indicating that it is largely mediated in a Smo-dependent manner (Fig. 4f,g). Using *Ptch1*^*Col1(het)*^ mice, treated with vismodegib, and harbouring an additional inducible tdTomato allele, we confirmed that *Col1a2CreER*-targeted cells remained exclusively stromal throughout the AOM/DSS protocol (Fig. 4h), showing that Hh activation remains confined to the stroma in this model.

The loss of one *Ptch1* allele had a marked effect on tumour development, yet it did not detectably influence the course of the DSS-induced colitis. Nevertheless, a role for Hh in modulating the immune infiltrate in the colon cannot be excluded and previous studies have suggested that stromal Hh signals can act as modifiers of the intestinal immune response^11^,^14^. To investigate the effect of Hh activation in a sporadic carcinoma model, we induced tumours in *Ptch1*^*Col1(het)*^ mice and controls with consecutive AOM injections in the absence of DSS^15^ (Fig. 4i). In accordance with AOM/DSS-treated mice, stromal Hh activation attenuated tumour development in this model (Fig. 4j,k).

Collectively, our results indicated that Hh-driven stromal signals can counteract the neoplastic transformation of overlying epithelial cells.

### Diminished BMP inhibitor expression upon Hh activation

To investigate the functional consequences of stromal Hh activation in greater depth, we performed gene expression analysis on colonic tissue from *Ptch1*^*ΔCol1*^ mice, in which one pulse of Tam induces homozygous inactivation of *Ptch1* in the stromal compartment, resulting in activation of Hh signalling in normal mucosa.

**Figure 5:**
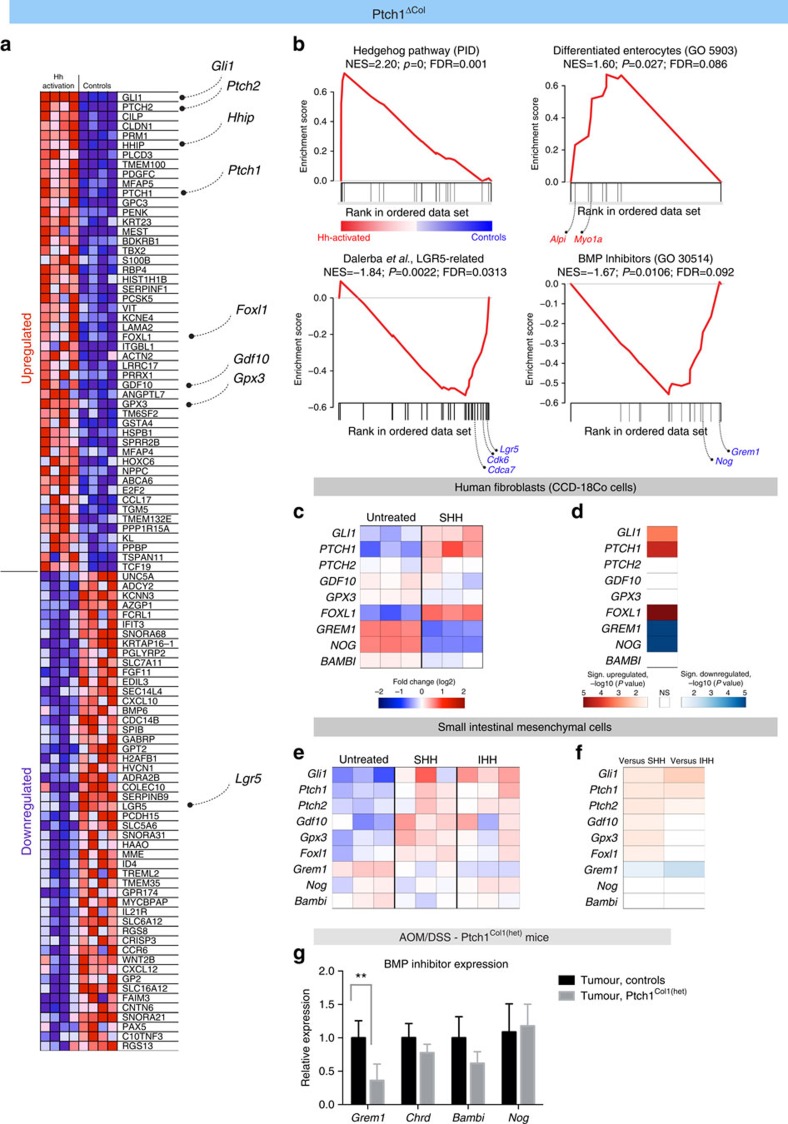
Transcriptional changes upon Hh activation. (**a**) Heatmap of the 50 highest ranked genes (using the GSEA algorithm^58^) in the murine colon upon knockout of stromal *Ptch1* in *Ptch1*^*ΔCol1*^ mice (*n*=4) versus controls lacking *Cre* recombinase (*n*=4) 7 days after a single dose of 5 mg Tam i.p. (**b**) GSEA indicates activation of canonical Hh signalling (left upper panel); enrichment of genes associated with enterocyte differentiation (right upper panel, gene ontology (GO) term, GO:0005903) and downregulation of colonic stem cell-associated genes (left lower panel) in parallel with loss of secreted BMP inhibitors (right lower panel). Normalized enrichment score (NES), *P*-value and fdr are indicated in each panel; gene lists are presented in the Supplementary Material. (**c**) Gene expression of indicated transcripts in the human colon fibroblast cell line, CCd-18Co, treated with 1 μg ml^−1^ SHH or vehicle for 72 h (data derived from GEO29316 (ref. 33)). (**d**) Illustration of significant changes in gene expression (fdr adjusted); note highly significant downregulation of *GREM1* and *NOG*, and upregulation of *FOXL1*. (**e**) Gene expression in cultured intestinal mesenchyme from E18.5 mouse embryos treated with Ihh or Shh amino-terminal polypeptide (2.5 μg ml^−1^) or vehicle for 24 h (data derived from GEO17840); scale as in **c**. (**f**) Illustration of significant changes in gene expression (fdr adjusted); *Grem1* was downregulated by both Shh and Ihh treatment; upregulation of *Gdf10*, *Gpx3* and *Foxl1* was significant in Shh-treated mesenchyme. Scale as in **d**. (**g**) mRNA expression of the indicated transcripts in tumours of *Ptch1*^*Col1(het)*^ mice (*n*>5) and tumours from control mice (lacking *Cre* recombinase, floxed *Ptch1* alleles or both; *n*>8); *P*-values from *t*-tests with fdr correction indicated, *P*=0.001 (*Grem1*), *P*=0.558 (*Nog*), *P*=0.603 (*Chrd*), *P*=0.206 (*Bambi*) and **P<0.01.

Gene expression data confirmed the upregulation of Hh targets (false discovery rate (fdr)=0.001), dominated by *Gli1*, *Ptch1*, *Ptch2*, and *Hhip* (Fig. 5a,b upper left panel and Supplementary Tables 1 and 2). Gene set enrichment analysis (GSEA) indicated a significant enrichment of transcripts associated with enterocyte differentiation (fdr=0.086), such as intestinal alkaline phosphatase (*Alpi*) or the brush border myosin Ia gene (*Myo1a*) (Fig. 5b upper right panel and Supplementary Table 3).

By contrast, Hh activation led to reduced expression of colonic stem cell-associated genes (fdr=0.0313), including *Lgr5*, *Cdca7* and *Cdk6*, all of which are restricted to the stem cell compartment at the crypt base (Fig. 5b lower left panel and Supplementary Table 4)^28^. ISH confirmed the reduced expression of *Lgr5*, whereas *Gli1* was increased as expected (Supplementary Fig. 7a,b). These effects of augmented stromal Hh signalling were paralleled by a marked reduction in secreted BMP inhibitors such as Gremlin 1 (*Grem1*) and Noggin *(Nog)* (fdr=0.092) (Fig. 5b lower right panel and Supplementary Table 5). Interestingly, *Gdf10* (also known as *Bmp3b*), a member of the BMP family exhibiting Hh-dependent expression, at least in the brain^29^, was among the top 50 upregulated genes (Fig. 5a).

Stroma-specific Hh activation entailed further changes, including alterations in known modifiers of intestinal tumourigenesis. For example, the mesenchymal factor, *Foxl1*, which was upregulated in *Ptch1*^*ΔCol1*^ mice (Fig. 5a), is directly controlled by Gli proteins^30^ and attenuates tumour development in a small intestinal adenoma model^31^. As a further example, the gene encoding plasma glutathione peroxidase (*Gpx3*), which has a protective effect in the AOM/DSS model^32^, was similarly upregulated (Fig. 5a).

To identify genes that show robust alterations in expression as a result of Hh activation specifically in stromal cells, we analysed two independent data sets: (i) human colonic fibroblasts (Fig. 5c,d) and (ii) murine intestinal mesenchymal cells (Fig. 5e,f), treated with Hh ligands *in vitro* (based on the GEO data sets GSE17840 and GSE29316 (refs 14, 33)). In both cases, activation of canonical Hh signalling was paralleled by significant upregulation of *Foxl1/FOXL1* and significant downregulation of the BMP inhibitor *Grem1/GREM1* (Fig. 5c–f). Other BMP inhibitors such as *Nog/NOG*, *Chrdl1/CHRDL1* and *Sostdc1/SOSTDC1* were significantly downregulated in one of the data sets, but not in both (Supplementary Table 6). Based on these data, we revisited tumours from *Ptch1*^*Col1(het)*^ mice, in which *Gli1* expression is increased (Fig. 4d), and analysed the expression of selected BMP inhibitors using real-time quantitative PCR. In line with the *in vitro* studies, we found that *Grem1* expression is downregulated in tumours from *Ptch1*^*Col1(het)*^ mice compared with controls (Fig. 5g).

Together, the data indicated that stromal Hh activation leads to complex transcriptional changes with a prominent, but not exclusive, role for the modulation of BMP signalling. These changes result in restriction of the colonic stem cell state signature and induction of epithelial differentiation markers.

### Hh activation blocks the growth of established tumours

AOM/DSS treatment of *Ptch1*^*ΔCol1*^ mice led to severe morbidity and mortality, precluding their use for the study of tumour initiation (Supplementary Fig. 7c,d,e). Without AOM/DSS treatment, *Ptch1*^*ΔCol1*^ mice had to be killed several weeks after Tam treatment, as they developed large, cystic stromal tumours predominantly at the ileoceacal junction, which occasionally ruptured or led to gross abdominal distension (Supplementary Fig. 7f,g). Similar tumours have been described in a related model, in which *Ptch1* was specifically inactivated in a more restricted stromal compartment^34^.

**Figure 6:**
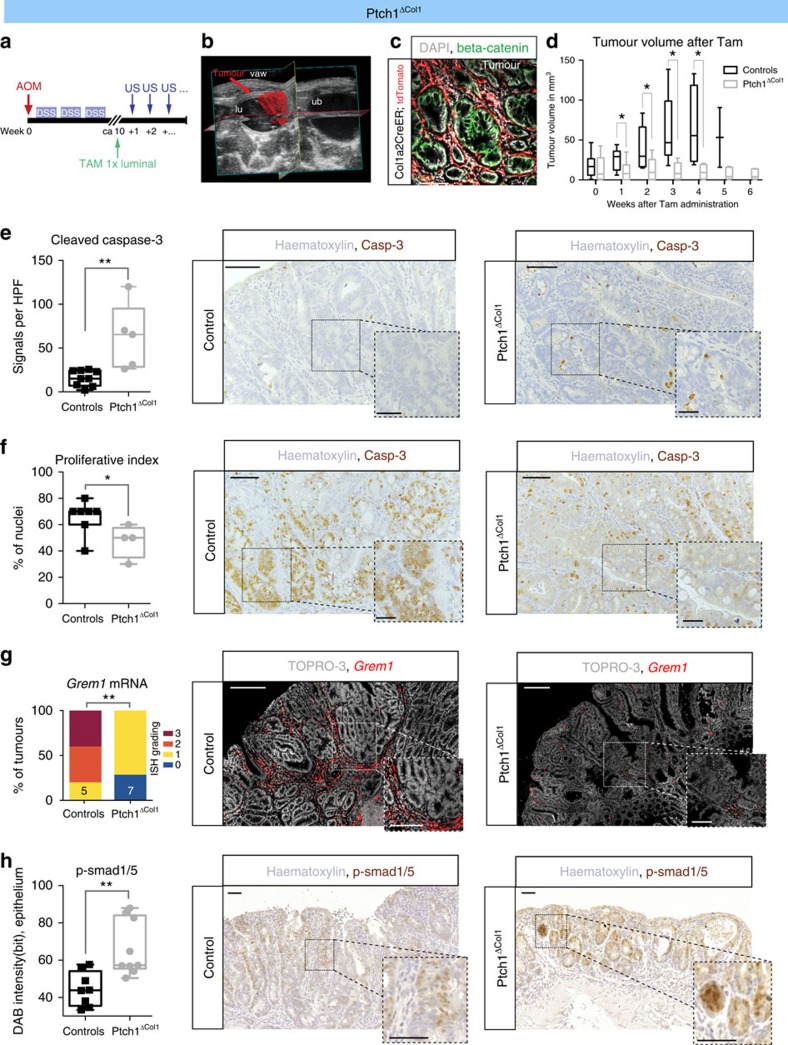
Growth arrest of established murine tumours upon stromal Hh activation. (**a**) Protocol schematic. (**b**) Example of a 3D μUS reconstruction; tumour volume (arrow) is highlighted in red, other hypoechoic (black) structure is the urinary bladder (ub); lu: colonic lumen, filled with US gel; vaw: ventral abdominal wall. (**c**) Tomato^+^ cells in an AOM/DSS-induced tumour from a *Col1a2CreER;R26-LSL-tdTomato* mouse 3 days after Tam administration; β-catenin (green) marks tumour cells; nuclear stain: 4,6-diamidino-2-phenylindole (DAPI); scale bar, 100 μm. (**d**) Tumour size as measured by μUS after Tam instillation (week 0). A total of *n*=5 *Ptch1*^*ΔCol1*^ mice with *n*=11 tumours and *n*=7 controls (littermates lacking *Cre* recombinase, floxed *Ptch1* alleles or both) with *n*=12 tumours are included; 1 to 5 tumours per mouse were analysed. The adjusted *P*-values (fdr) from *t*-tests are indicated: *P*=0.406 (week 0), *P*=0.022 (week 1), *P*=0.029 (week 2), *P*=0.026 (week 3), *P*=0.026 (week 4) and *P*=0.078 (week 5). Box-and-whisker plots: whiskers represent minima and maxima. In the control group, mice had to be killed because of bleeding per anus, leaving 2 tumours for US analysis in week 5 and none in week 6. (**e**) Quantification of cleaved caspase-3 positivity per high-power field (original magnification × 400): controls (*n*=9 tumours), *Ptch1*^*ΔCol1*^mice (*n*=5 tumours), *P*=0.0034 (*t*-test). Middle and right panels show representative IHC images for similar-sized tumours 3 weeks after Tam treatment for the indicated genotypes. Scale bars, 20 μm. (**f**) Proliferative index as a percentage of Ki67^+^ nuclei for *n*=7 controls and *n*=4 tumours from *Ptch1*^*ΔCol1*^ mice, *P*=0.0467 (*t*-test) and representative images (middle and right panels). Scale bars, 50/20 μm. (**g**) ISH semi-quantification of *Grem1* in control mice (*n*=5 tumours) and *Ptch1*^*ΔCol1*^mice (*n*=7 tumours). Stacked bars represent the frequency of intensity grading; *P*=0.0076 (Mann–Whitney test), middle and right panels show representative images for the two groups; scale bars, 200/100 μm. (**h**) Quantification of p-Smad1/5 IHC in control mice (*n*=8 tumours) and *Ptch1*^*ΔCol1*^ mice (*n*=9 tumours); *P*=0.0028 (*t*-test), middle and right panels show representative images for the two groups; scale bars, 50 μm; (**e**–**h**) boxed areas show magnified regions. *P<0.05, **P<0.01 and ***P<0.001.

Nevertheless, we were able to induce tumours with AOM/DSS in *Ptch1*^*ΔCol1*^mice that had not received Tam previously and we used this model to activate Hh signalling in established tumours. It has recently been shown that high levels of Wnt signalling are necessary for tumour maintenance, and that restoration to homeostatic Wnt levels causes tumour regression^21^. This prompted us to ask whether activated stromal Hh signalling might restrain tumour growth in a similar manner. Once AOM/DSS-induced lesions of similar sizes were detected, we used three-dimensional (3D) μUS to monitor tumour volumes over time in *Ptch1*^*ΔCol1*^ mice and control mice after Tam administration (Fig. 6a,b). Using *Col1a2CreER;R26-LSL-tdTomato* mice, we confirmed that tdTomato^+^ cells were directly adjacent to the tumour cells (Fig. 6c).

One week after Tam treatment, delayed growth was evident in tumours from *Ptch1*^*ΔCol1*^ mice compared with littermate controls (Fig. 6d). Over the course of several weeks, tumours with Hh-activated stroma frequently entered permanent macroscopic growth arrest or, in the case of smaller lesions, showed regression, whereas tumours in control animals progressed (Fig. 6d and Supplementary Movies 1 and 2). IHC analysis of cleaved caspase-3 (Fig. 6e) and Ki67 (Fig. 6f) indicated increased tumour cell apoptosis and diminished proliferative activity, respectively, following stromal Hh activation.

To assess whether *Grem1* is reduced as a consequence of stromal Hh activation in the tumours, we used RNA ISH and semi-quantitatively evaluated the staining intensity^35^. Although *Grem1* was strongly expressed in the tumour stroma of control mice, expression was significantly reduced in *Ptch1*^*ΔCol1*^ mice (Fig. 6g). In addition, tumour-epithelial p-Smad1/5 staining was consistently more abundant in tumours from *Ptch1*^*ΔCol1*^ mice, indicating increased epithelial BMP downstream activity as a result of the augmented stromal Hh signal (Fig. 6h).

### Diminished downstream Hh response in human colon cancer

**Figure 7:**
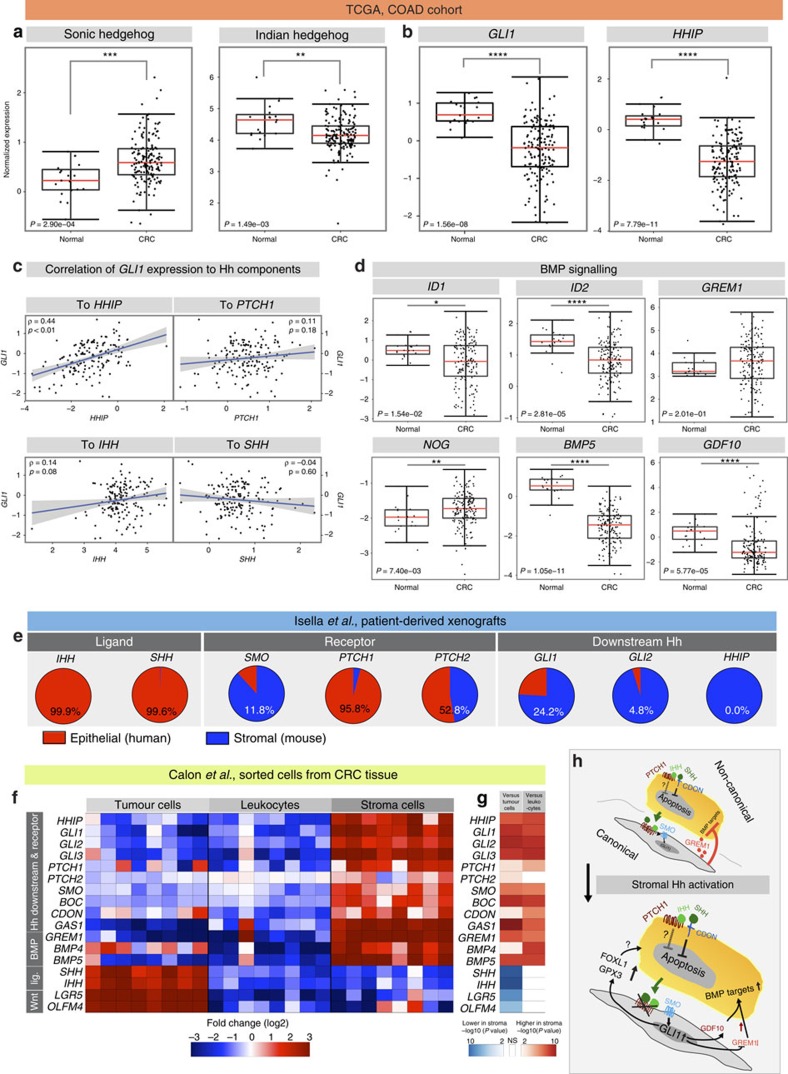
Diminished Hh signalling in human colonic adenocarcinomas. (**a**) TCGA analysis (box plots) of Hh ligand expression and (**b**) expression of Hh downstream targets in 155 human colon carcinomas; *P*-values (Wilcoxon rank-sum test) are indicated in the panels. (**c**) Correlation between *GLI1* expression and expression of *HHIP*, *PTCH1*, *IHH* and *SHH*; correlation coefficients and *P*-values are given in the panels. (**d**) Expression of the BMP targets *ID1* and *ID2*, the BMP inhibitors *GREM1* and *NOG*, and the BMP ligands *BMP5* and *GDF10*. *P*-values are indicated in the panels. (**e**) Analysis of Hh pathway expression in patient-derived xenografts, based on bi-species RNA-sequencing data^37^. Hh ligands are expressed exclusively by human tumour cells, whereas transcripts of downstream targets are mostly of murine origin (that is, stromal). Expression of *SMO* and *PTCH1*, as well as its homologue *PTCH2*, is seen in both compartments (middle panel). (**f**) Illustration of gene expression in human CRC (*n*=8) sorted for tumour/epithelial cells (Epcam^+^, CD45^−^), leukocytes (Epcam^−^, CD45^+^) and fibroblasts (double negative). Each column represents expression data from an individual patient for each compartment (based on GEO data set GSE39395). (**g**) Differential expression of the indicated transcripts in comparison with stromal expression. Red/blue cells indicate significant up-/downregulation in the stroma; fdr<0.05. (**h**) Model of Hh signalling in CRC: fibroblasts (grey) express the Hh receptors, PTCH1 and SMO, and respond to tumour cell-derived ligand by activation of canonical Hh signalling. Reduced stromal Hh aticvity is associated with paracrine inhibition of BMP signalling, partly mediated via derepression of GREM1. Tumour cells (orange) are largely incapable of canonical Hh signalling. However, the Hh co-receptor CDON and possibly PTCH1 itself may function as dependence receptors for the ligands, IHH and SHH, and inhibit tumour cell apoptosis. Upon activation of canonical Hh signalling in the stroma, for example, by knockout of *PTCH1* (‘X', lower panel), expression of BMP inhibitors is repressed, whereas expression of BMP ligands such as *GDF10* is upregulated, cumulatively leading to enhanced BMP signalling in the tumour cells. Other stromal factors, such as FOXL1 and GPX3, may contribute independently of BMP signalling to the protective effect of active stromal Hh signalling. *P<0.05, **P<0.01, ***P<0.001 and ****P<0.0001.

Based on the unexpected expression patterns of Hh pathway members in murine tumours, we decided to re-assess Hh expression in human CRC in more detail, including the pathways' downstream targets. To this end, we first analysed microarray gene expression data from The Cancer Genome Atlas (TCGA), representing 155 colon cancer patients^2^. Although *SHH* was upregulated in carcinomas as previously reported^3^, *IHH* was moderately downregulated (Fig. 7a). As in the mouse model, the downstream targets, *GLI1* and *HHIP*, were downregulated (Fig. 7b) and, strikingly, no correlation between *GLI1* and Hh ligand expression was observed (Fig. 7c). In addition, we found that the BMP targets, *ID1* and *ID2*, were significantly downregulated in the carcinomas, whereas *GREM1* and *NOG* expression increased, albeit not significantly in the case of *GREM1* (Fig. 7d). Moreover, expression of the BMP ligands, *GDF10/BMP3b* and *BMP5*, was reduced significantly (Fig. 7d).

In some colon cancer cell lines with mutations in *KRAS* or *BRAF*, cancer cell-autonomous *GLI1* activation has been described^36^. However, we found no correlation between the presence of mutated *KRAS* or *BRAF* and *GLI1* mRNA levels (Supplementary Fig. 8a). Instead, there was a weak but significant correlation between mutations in the *APC* gene and low *GLI1* levels (Supplementary Fig. 8a).

*GLI1* mRNA levels correlated well with stromal gene expression signatures in the TCGA data set (Supplementary Fig. 8b), indicating that activation of downstream Hh signalling is largely confined to the stromal compartment. To confirm this further, we probed gene expression data from CRC patient-derived xenografts, in which murine stromal cells replace the human stroma after transplantation (based on GEO GSE56710 and GSE35144 data sets)^37^. In the xenografts, expression of the ligands, *SHH* and *IHH*, was exclusively epithelial, whereas downstream signalling was largely restricted to the stroma, as transcripts of *GLI1* (>75%) and *GLI2* (95%), as well as *HHIP* (100%) were predominantly or exclusively murine (Fig. 7e).

To assess gene expression in the different cellular compartments in a more quantitative manner, we analysed Hh and BMP pathway components in CRCs sorted for epithelial cells (CD45^−^, Epcam^+^), leukocytes (CD45^+^, Epcam^−^) and stromal cells (CD45^−^, Epcam^−^; based on the GEO39395 data set)^38^. We found a similar pattern to that in the xenografts, such that downstream Hh targets were enriched in the stroma to a highly significant degree, whereas ligand expression was epithelial (Fig. 7f,g). *GREM1* and the BMP ligand *BMP5* were enriched significantly in the stromal fraction, in keeping with the results from the mouse model, whereas tumour cells expressed *BMP4* in several cases. Of note, the Hh co-receptor *CDON* and *PTCH1* were expressed in the tumour cells at a level similar to that seen in the stroma, although this did not lead to increased expression of Hh downstream targets in the epithelial compartment (Fig. 7f).

In summary, the results obtained from different CRC cohorts demonstrated that human colon cancer exhibits a marked decrease in downstream Hh target gene expression, which is predominantly, if not exclusively, of a stromal nature.

## Discussion

Under homeostatic conditions, the mode of intestinal Hh signalling is exclusively paracrine, from the ligand-secreting epithelium to the receptive underlying stroma^11^,^39^. However, a role for cell-autonomous downstream Hh activity in colon cancer cells has been suggested, in particular at later tumour stages, in which Hh activation drives metastasis^4^,^40^.

In the current study, our analyses of stromal and epithelial gene expression strongly suggest that there is little, if any, cell-autonomous Hh downstream activity in colon cancer cells (Fig. 7e,f). Together with the marked overall downregulation of *GLI1* and *HHIP* (Fig. 7b), these results provide consistent evidence that stromal downstream Hh activity is diminished in colon cancer. However, as some CRC cell lines are dependent on *GLI1* (refs 36, 40), it is possible that downstream Hh signalling might play a role in a tumour cell subpopulation. On the other hand, most CRC cell lines do not express a complete set of Hh downstream targets^41^. Aside from a role for canonical Hh signalling, it has previously been suggested that Hh ligands can prevent colon cancer cells from entering apoptosis by binding to the dependence receptor, CDON, in an autocrine manner (independent of the role of CDON as a Hh co-receptor)^42^. As we find that *CDON* is expressed in the tumour cell compartment (Fig. 7f), this leaves a possible tumour-promoting role for a non-canonical SHH/CDON/PTCH1 axis, acting independently of downstream Hh signalling (Fig. 7h).

In contrast to carcinomas, human colonic adenomas from patients with familial adenomatous polyposis as well as small intestinal adenomas from *Apc*^*lox15*^ mice exhibit increased levels of *IHH/Ihh* and *GLI1/Gli1* (ref. 12). In *Apc*^*lox15*^ mice the presence of *Ihh* is required for adenoma development^12^, whereas in the AOM/DSS model *Ihh* loss fuels tumourigenesis (Fig. 2a). Importantly, treatment of later stage small intestinal murine adenomas with Hh inhibitors promoted, rather than restricted, tumour growth in the *Apc*^*min*^ model^12^. Almost all functional data on intestinal Hh signalling from *in vivo* models have thus far been acquired in the small intestine^11^,^12^,^13^,^14^,^43^. However, the tissue architecture and cellular composition of the small intestine differ considerably from those of the colon, in which the vast majority of human intestinal cancers arise. It is therefore noteworthy that colonic tumours in mice, whether they are induced chemically with AOM/DSS (Fig. 1), or genetically, as in the ShApc model^21^ (Supplementary Fig. 2), recapitulate the Hh expression pattern seen in human CRCs^12^. Hence, tissue-specific properties of the small intestine and colon may explain the different requirements in the two locations with regard to dependence on Hh signalling.

Across various colon cancer mouse models and in human CRC, downstream Hh activity can be lost despite increased ligand expression (Figs 1 and 7, and Supplementary Fig. 2). This might be the result of alterations in stromal gene expression programmes that render stromal cells indifferent to the epithelial ligand^44^. An alternative explanation has recently been proposed by Shyer *et al*.^45^, who found that during development Hh ligands are concentrated at the villus tips merely as a result of the physical folding of the epithelium. This leads to Hh activation in the stroma and differentiation of the epithelium at the villus tips^45^. It is tempting to speculate that, as a result of perturbations in the tissue architecture during tumourigenesis, ligand concentrations are insufficient to activate downstream Hh signalling in the tumour stroma. Interestingly, in the Apc-shRNA model, the expression of *Gli1*, *Gli2*, and *Hhip* is consistently reduced further in tumours carrying an additional mutation in *Kras* (Supplementary Fig. 2c), which show higher levels of dysplasia than tumours in Apc-shRNA animals, as well as invasive behaviour, supporting a model in which disrupted tissue architecture interferes with ligand availability and induction of the stromal Hh response^21^. Moreover, in our hands, AOM/DSS-induced tumours occasionally displayed signs of invasion through the *muscularis mucosae* (Supplementary Fig. 9 and refs 16, 46).

Although the AOM/DSS model recapitulates the Hh expression pattern of human CRC as well as parts of the mutagenic landscape of sporadic human colon cancers^16^, tumour development is accelerated by colitis, which is not a self-evident feature of most human carcinomas. Nevertheless, sporadic colorectal carcinogenesis is characterized by, and is dependent on, a complex inflammatory cell infiltrate and immunocompromised mice are protected from both inflammatory-associated and sporadic colon tumours^47^,^48^. On a clinical note, our data indicate that caution is warranted particularly when using Hh antagonists in patients with inflammatory bowel disease, who are at an increased risk of developing CRC^49^. However, together with data from pancreatic and bladder cancer, in which Hh antagonism similarly accelerates carcinogenesis^50^,^51^, our results point to the possibility that treatment with Hh antagonists could increase the risk for several solid malignancies, including CRC.

Functionally, the data suggest a dominant, but not exclusive role for BMP modulation as a mediator of stromal–epithelial crosstalk. BMP signalling is active in adenomas, but is downregulated in CRCs^52^ (Fig. 7d) and thus mirrors the expression pattern of downstream Hh effectors. In sporadic CRC, high *GREM1* expression is associated with treatment resistance and inferior survival^53^. Moreover, ectopic epithelial expression of GREM1 causes hereditary mixed polyposis syndrome, which is characterized by the appearance of multiple colonic polyps that frequently progress to carcinomas^54^. This can be modelled in mice, where ectopic Grem1 expression leads to the reacquisition of stem cell properties in differentiated cells^53^, which are consequently able to initiate intestinal neoplasia. We show that, as a consequence of Hh activation, attenuated BMP inhibitor expression is conversely associated with diminution of the intestinal stem cell signature (Fig. 5a,b). Thus, it seems plausible that loss of stromal BMP inhibitors due to the activation of stromal Hh signalling (Fig. 5b) may have the potential to restrain colonic tumour initiation and progression (Fig. 7h).

The importance of paracrine Hh signalling in tumour–stroma cross-talk has been recognized for more than a decade—yet accumulating data now indicate that Hh-driven signals might represent exploitable targets that are not limited to colon cancer therapy, but extend to other cancer types.

## Methods

### Mice

C57BL/6 mice (referred to as wt) were obtained from Scanbur, Sweden, and used at >8 weeks of age. All mice were on a C57BL/6 background. *Gli1*^*lacZ/+*^ and *GliCreER*^*T2*^ mice were a kind gift from Fritz Aberger, whereas *R26-LSL-tdTomato* (*B6.Cg-Gt[ROSA]26Sortm14[CAG-tdTomato]Hze/J*) and *Col1a2CreER* (*B6.Cg-Tg[Col1a2-cre/ERT]7Cpd/J*) mice were obtained from the Jackson Laboratory (Bar Harbor, USA). *Ptch1*^*fl/+*^ mice have been described previously^27^. For *IhhΔ*^*Vil*^ mice, *VillinCreER*^*T*2^ mice were crossed with *Ihh*^*flox/flox*^ mice and *Rosa26-ZSGreen* mice, to generate *VillinCreERT2-ZsGreen-Ihh*^*flox/flox*^ animals.

Mice receiving DSS were assessed daily in a cumulative score until recovery (weight, movement and body posture, piloerection, skin condition, food consumption, excretions and breathing). Littermates were used as controls and experimental groups were housed in one cage wherever possible. In the case of *Cre/Lox-p* models, the control animals lacked either the *Cre* or floxed alleles or both.

### Chemical models of CRC

AOM (Sigma-Aldrich, A5486) at 12.5 mg kg^−1^ bodyweight was administered intraperitoneally (i.p.) on day 0 and DSS (DB001; Tdb Consultancy, Sweden) was initiated at a concentration of 2% on day 5 and was administered for 5 days^15^. DSS cycles were repeated twice with 14 days of normal drinking water in between. All DSS preparations were renewed after 2 days. For the sporadic model, AOM was administered i.p. once a week at 12.5 mg kg^−1^ body weight for 10 consecutive weeks.

For *IhhΔ*^*Vil*^ mice specifically, 10-week-old animals were injected i.p. with 1 mg Tam (T5648; Sigma-Aldrich) for 5 consecutive days. Two weeks after Tam initiation, mice were injected with AOM (10 mg kg^−1^) and received drinking water supplemented with 1.75% DSS for 4 days, which was then changed to untreated drinking water. During weeks 5 and 8, the mice received 1.25% DSS for 4 and 3 days, respectively. The DSS concentration and treatment duration were adjusted due to augmented weight loss. After 12 weeks, the mice were killed.

### Vismodegib and Tam treatment

Vismodegib (V-4050, LC Laboratories) was administered i.p.^55^,^56^ in dimethyl sulfoxide twice daily at 25 mg kg^−1^ body weight for 6 days per week.

For intracolonic administration, 4OH-Tam (H6278; Sigma-Aldrich) was dissolved in 99.5% ethanol, vortexed, further diluted in corn oil (S5007, Sigma-Aldrich) to reach the working concentration and sonicated for 30 min at 37 °C in a water bath. The solution was kept at 37 °C in darkness until it was instilled intraluminally at 1 mg 25 g^−1^ bodyweight. Instillation was performed with US guidance under isoflurane anaesthesia (described below). For visualization of the instilled substance with microcomputed tomography (μCT) imaging, 0.1 mg ml^−1^ barium sulphate (Barium Sulphate (#11381499) 97%, Alfa Aesar) was added to the Tam/oil suspension. The μCT was performed under isoflurane anaesthesia (see below) using a Quantum FX μCT imaging system (Caliper Life Sciences, Perkin Elmer, Inc.) a few minutes after administration of the Tam/oil/barium mixture.

For systemic labelling experiments in *Col1a2CreER;R26-LSL-tdTomato* and *Gli1CreER*^*T2*^*;R26-LSL-tdTomato* mice, 5 mg of Tam was dissolved in corn oil using sonication at 37 °C at 20 mg ml^−1^ and injected once i.p.

### Ultrasound

The Vevo 2100 system (Visualsonics, Canada) was used for high-frequency US^57^. Mice were anaesthetized with 1–2% v/v isoflurane (Baxter, KDG9621). Hair over the abdomen was removed using a depilatory cream. The mice were scanned in a supine position on a heating plate (40 °C) with constant cardiovascular monitoring and protective eye ointment. Scans were performed at 40 MHz (probe MS550D; axial resolution 40 μm) using a 3D-motor for *z* axis resolution^57^. For administration of intraluminal contrast during colonic imaging, a plastic gavage tube was advanced carefully via the anus and small amounts of US gel were injected to inflate the colonic lumen gently under US surveillance. The largest tumour cross-sectional area was determined visually. For 3D scanning, the *z* resolution was set to 76 μm and the whole tumour length was scanned. Tumour outlines were drawn manually for each slice and 3D reconstruction was performed using Visualsonics Vevo software (v1-5.0).

### Immunofluorescence

For IF, the colon was removed, faeces were gently washed out with cold PBS and the tissue was immediately frozen on dry ice in Tissue-Tek O.C.T. compound and stored at −80 °C. Sections were cut using a cryotome at 20–100 μm and incubated for 30 min in PBS with 0.5% Triton X-100 (Sigma-Aldrich; T8787) and 0.1% TOPRO-3 (Invitrogen; T3605). Slides were mounted between two coverslips (Menzel, Nr. 1.5 (0.16–0.19 mm)) and scanned using a Zeiss LSM710 confocal microscope in single-photon mode. For antibody staining, 20–100 μm sections were cut in a cryotome and incubated overnight in antibody diluted in PBS plus 0.5% Triton-X-100, 0.5% BSA and 0.5% dimethyl sulfoxide at 4 °C. A species-appropriate secondary antibody (1:250 dilution) was chosen from Alexa Fluor dyes (Invitrogen) using an appropriate wavelength depending on the primary antibody and the presence of endogenous fluorophores. The antibodies and dilutions were as follows: anti-vimentin (Santa Cruz, sc7557, 1:500); anti-desmin (Abcam, ab8592, 1:500); anti-α-sma (Abcam, ab5694, 1:500); anti-collagen1 (Abcam, ab34710, 1:200); anti-β-catenin (Cell Signalling, #9582, 1:100); anti-Ita6 (CD49f, BD Pharmigen, # 555734, 1:100).

### IHC and X-gal staining

For IHC, the colon was incised longitudinally and fixed in 4% paraformaldehyde (PFA) in PBS overnight at 4 °C before routine processing and paraffin embedding. Next, 4 μm sections were baked at 60 °C for 1 h and rehydrated.

The sections were subjected either to a heat-induced epitope retrieval step or to proteinase-induced epitope retrieval, depending on the primary antibody used. Heat-induced epitope retrieval was performed in citrate buffer (pH 6) or DIVA reagent (Biocare Medical, DV2004) using a pressure cooker. Protein-induced epitope retrieval was performed in a 37 °C water bath using 0.05% protease (Sigma). Sections were incubated with antibodies against B220 (eBioscience; clone RA3-6B2; 1:200), CD3 (Dako; #N1580; 1:20), F4/80 (Invitrogen; clone BM8; 1:100), MPO (Dako #A0398; 1:500), β-catenin (Cell Signalling; #9582; 1:100), phospho-smad1/5 (Ser463/465; Cell Signalling; 9516S; 1:50), cleaved caspase-3 (Asp175; Cell Signalling; 9661S; 1:200) or Ki67 (Novocastra; NCL-Ki67p; 1:200) for 1 h at room temperature (RT). After rinsing, the sections were incubated with a biotinylated secondary antibody: donkey anti-rabbit, rabbit anti-rat antibody (Dianova) or rabbit anti-goat (Vector, BA-5000), followed by incubation with alkaline phosphatase-labelled streptavidin (Dako; CD3, B220, F4/80, MPO, β-catenin) for 30 min at RT or streptavidin peroxidase (Invitrogen; for all others). Alkaline phosphatase was revealed with Fast Red (Dako; AR17911-2) and peroxidase was revealed with DAB (Invitrogen; DAB plus reagent kit; #002020). Sections were counterstained with haematoxylin or eosin (for X-gal stained tissue) and slides were mounted with water-based mounting medium (Polyscience; #18606). Negative controls omitted the primary antibodies. Triple-IF was performed with antibodies against desmin (AF3844, R&D, 1:200) and vimentin (#5741, Cell Signalling, 1:100), followed by secondary antibodies as described above together with a Cy3-coupled anti-sma antibody (C6198, Sigma Aldrich, 1:200).

X-gal staining was performed as previously described with slight modifications^27^. Briefly, colon tissue was fixed in 2% PFA/PBS with 0.2% v/v glutaraldehyde for 30 min at RT. Tissues were washed once for 15 min in 2 mM MgCl in PBS plus 0.01 % Nonidet P-40 (BDH Laboratory Supplies; #56009). Tissues were incubated for 15 h at 37 °C in X-gal substrate solution (stock solution: 100 ml of washing buffer, 1 ml of 5 mM potassium ferricyanide, 1 ml of 5 mM potassium ferrocyanide and 2.5 ml of 40 mg ml^−1^ X-gal (Sigma, B9285) in *N*,*N*-dimethylformamide). Specimens were washed once in PBS, fixed in 4% PFA/PBS for 4 h at RT and paraffin embedded.

Microphotographs were taken using a Leica DMLB microscope with a Leica DC300F camera or with a Panoramic MIDI scanner (3DHISTECH), imported with PanoramicViewer software (v.1.15.4). Adobe Photoshop (CS5, v12.1) was used to adjust brightness and contrast. Changes were always applied to the whole image.

The colour deconvolution plug-in for ImageJ was used to quantify DAB and X-gal intensities, and to manually define regions of interest. ‘Periphery' was defined as the outermost area with the strongest X-gal staining. Regions were drawn manually and the pixel intensities for DAB (brown) and X-gal (blue) were quantified on an 8-bit scale.

### RNA *in situ* hybridization

The RNAScope 2.0 High Definition Kit (DAB) (#310035) and the Multiplex Fluorescent Kit (#320850) were used^35^. The following probes were all from Advanced Cell Diagnostics: *Mus musculus* leucine-rich repeat containing G protein coupled receptor 5 (*Lgr5*), #312171; mRNA *M. musculus* GLI-Krüppel family member GLI1 (*Gli1*), mRNA, #311001; *M. musculus Ihh* mRNA, #413096; *M. musculus Grem1* mRNA, #314741; *M. musculus* Ptch1 *mRNA*, #402811. Hybridization was performed according to the manufacturer's protocol with slight modifications (pre-treatment 2 was prolonged to 20 min and the developing time was extended to 15 min). Expression was assessed semi-quantitatively as suggested by the manufacturer from 0 (absent), 1 (sporadic positive cells with single spots), 2 (frequent spots with occasional clusters of signal) to 3 (abundant spots and clusters).

### Real-time quantitative PCR

A 20–30 mg sample of snap-frozen tissue was disrupted using a VDI 12-homogenizator (VWR International) and mRNA was extracted according to the manufacturer's protocol using an RNeasy midi kit (Qiagen, #75142). Real-time PCR was performed using the 7500 Fast Real-Time PCR System (Applied Biosystems). Power Green PCR Master Mix (Applied Biosystems; #4368708) comprised SYBR Green I Dye, AmpliTaq Gold DNA Polymerase, dNTPs, buffer components and ROX as the passive reference dye. The following primers were used: glyceraldehyde-3-phosphate dehydrogenase (*Gapdh*), forward: 5′- GGTGTGAACGGATTTGGCCGTATTG -3′, reverse: 5′- CCGTTGAATTTGCCGTGAGTGGAGT -3′; *Rplp0/ARP*, ribosomal protein, large, P0, forward: 5′- TGCACTCTCGCTTTCTGGAGGGTGT -3′, reverse: 5′- AATGCAGATGGATCAGCCAGGAAGG -3′; GLI family zinc finger 1 (*Gli1*), forward: 5′- CGTTTAGCAATGCCAGTGACC -3′, reverse: 5′- GAGCGAGCTGGGATCTGTGTAG -3′; *Ihh*, forward: 5′- GGCTTCGACTGGGTGTATTA -3′, reverse: 5′- CGGTCCAGGAAAATAAGCAC -3′; *Ptch1* forward: 5′- TTGGGATCAAGCTGAGTGCTG -3′, reverse: 5′- CGAGCATAGCCCTGTGGTTCT -3′; *Shh* forward: 5′- TGGAAGCAGGTTTCGACTGG -3′, reverse: 5′- GGAAGGTGAGGAAGTCGCTGT -3′. *Gli2* forward: 5′- TGAGGAGAGTGTGGAGGCCAGTAGCA -3′, reverse: 5′- CCGGGGCTGGACTGACAAAGC -3′; *Hhip* forward: 5′- TAACGGCCCTTTGGTTGGTGGATTT -3′, reverse: 5′- AGCAAAGCCCAGTGACCAAGCAATG -3′; *Lgr5* forward: 5′- CCAATGGAATAAAGACGACGGCAACA -3′, reverse: 5′- GGGCCTTCAGGTCTTCCTCAAAGTCA -3′; *Axin2* forward: 5′- CAGGAGGATGCTGAAGGCTCAAAGC -3′, reverse: 5′- CTCAAAAACTGCTCCGCAGGCAAAT -3′; *Ihh* forward: 5′- GGCTTCGACTGGGTGTATTA -3′, reverse: 5′- CGGTCCAGGAAAATAAGCAC -3′; *Ptch2* forward: 5′- CCCGTGGTAATCCTCGTGGCCTCTAT -3′, reverse: 5′- TCCATCAGTCACAGGGGCAAAGGTC -3′; *Grem1* forward: 5′- AGACCTGGAGACCCAGAGTA -3′, reverse: 5′- GTGTATGCGGTGCGATTCAT -3′; *Nog* forward: 5′- AAGGATCTGAACGAGACGCT -3′, reverse: 5′- GCGAAGTAGCCATAAAGCCC -3′; *Chrd* forward: 5′- GATGCTGTTCCCACTGCAC -3′, reverse: GGCCCATCCTCTTGGTCATA -3′; and *Bambi* forward: 5′- CGCCACTCCAGCTACTTCTT -3′, reverse: 5′- TGAGCAGCATCACAGTAGCA -3′.

### Gene expression microarrays

RNA was isolated from 1 cm of fresh-frozen whole colonic tissue from *Col1a2CreER;Ptch1*^*fl/fl*^ mice (*n*=4) and controls (*n*=4) starting 1.5 cm proximal to the anus. RNA quality was assessed using the Agilent 2200 Tape Station system; RNA integrity numbers >8 were considered sufficient. Affymetrix Mouse Gene ST 2.0 arrays were used. Robust multi-array average median polish procedures were applied for normalization. For GSEA analyses^58^, the GSEA Java plug-in v2.1.0 was used to probe non-log-transformed normalized expression data with standard settings (with the exception that the permutation type was set to ‘gene_set' and gene sets <15 genes were permitted). Gene lists were derived either from publications as indicated, from the Molecular Signature Database (www.broadinstitute.org/gsea/msigdb), or from gene ontology searches via the AmiGO2 database v 2.1.4 (www.geneontology.org), curated to include ‘manual assertion' and ‘experimental evidence', and excluding ‘sequence similarity evidence' (see also Supplementary Tables 2–5 for the gene sets used).

### TCGA query

Processed microarray data for colon adenocarcinoma (AgilentG4502A_07_3) were analysed using gene expression data obtained from TCGA^2^ accessed via the UCSC Cancer Browser^59^. In total, the data set obtained included expression data for 155 primary tumours and 19 normal tissue samples. All analyses were performed in R (version 3.1.1). Associations between *GLI1* expression and the selected genes were evaluated using Spearman's rank correlation coefficient. The correlations were derived using the rcorr function from the Hmisc package. Differences in expression levels between tumours and normal tissue samples were analysed using the Wilcoxon rank-sum test from the R stats package. Pre-calculated ESTIMATE scores for the TCGA colon adenocarcinoma data were retrieved from the ESTIMATE web repository (http://ibl.mdanderson.org/estimate/)^60^.

### Analysis of GEO data sets

Data for the GEO data sets GSE67186, GSE39395, GSE17840 and GSE29316 were retrieved from the GEO website (http://www.ncbi.nlm.nih.gov/geo). For RNA-sequencing data (GSE67186), DESeq^61^ was used to normalize the raw count data and to test for differential expression comparing both the control (shRenilla) to each treatment group (shAPC and shAPC/Kras) and the treatment groups to each other. Microarray data from Affymetrix Human Genome U133 Plus 2.0 (GSE39395 and GSE29316) and Affymetrix Mouse Genome 430 2.0 arrays (GSE17840) were robust multi-array average normalized using the affy package^62^ and differential expression between treatment groups was analysed using limma^63^. For GSE39395, gene expression in stromal cells was compared with both epithelial cells and leukocytes, whereas in the cases of GSE29316 and GSE17840, Hh-ligand treated cells were compared with untreated or vehicle-treated controls. The expression pattern of *a priori* selected genes was subsequently analysed and differential expression was called based on an fdr< 0.05.

### Statistics

All data (except for microarray and sequencing data) were analysed using GraphPad Prism v6.0e and are presented as the mean and s.e.m. unless otherwise specified in the figure legends. Statistical tests are indicated in the figure legends. All *t*-tests were two-sided. The type 1-error rate was set to 5%, except for GSEA, for which results with an fdr <0.25 were considered significant as previously suggested^58^.

### Study approval

All animal experiments were performed in accordance with local ethical guidelines and with approval from the local ethics committee (the Swedish Board of Agriculture and the Animal Ethics Committee of the Amsterdam Medical Center, The Netherlands).

### Data availability

The gene expression data that support the findings of this study have been deposited at GEO (with the accession code GSE67172); expression data referenced in this study are available in GEO (GSE67186 (ref. 21), GSE39395 (ref. 38), GSE17840 (ref. 14) and GSE29316 (ref. 33)). All other data that support the findings of this study are available from the corresponding author (R.T.) upon request.

## Additional information

**How to cite this article:** Gerling, M. *et al*. Stromal Hedgehog signalling is downregulated in colon cancer and its restoration restrains tumour growth. *Nat. Commun.* 7:12321 doi: 10.1038/ncomms12321 (2016).

## Supplementary Material

Supplementary Information Supplementary Figures 1-9 and Supplementary Tables 1-6.

Supplementary Movie 1 μUS of a Ptch1ΔCol1 mouse prior to Tamoxifen administration Ultrasound (US) of a murine abdomen scanned from the anus rostral to the level of the liver. Note that this scan was performed by moving the US probe manually over the abdomen rather than using a 3D motor. The colonic lumen was gently inflated with US gel (peristaltic waves lead to variation in colon diameter). The colon is the dark (hypoechoic) ventral structure. From frame 209 to frame 231 (indicated at the bottom), a tumour appears on the lower left part of the colonic lumen (corresponding to the dorsal right side of the mouse). A very small mucosal irregularity occurring ventrally in the same area can be assumed. Frames 272 to approximately 330 depict a larger tumour. Here, the kidney is clearly visible as a landmark in the lower right part of the image (dorsal left side of the animal). From frames 359 onwards, a large tumour can be seen, that in additional directed scans did not appear connected to the aforementioned tumour. In the vicinity of this third tumour, liver tissue is visible (from frame 435 onwards). At this time point, Tam was administered (week 0, Fig. 6c).

Supplementary Movie 2 μUS of a Ptch1Col1 mouse after Tamoxifen administration The same mouse seen in Supplementary video 1, scanned 3 weeks after the first scan. Note that the tumours are not discernable in this scan, using the kidney (from approximately frame 200) or liver tissue (from frame 535 onwards) as landmarks. Further scans revealed that the third tumour was still visible.
