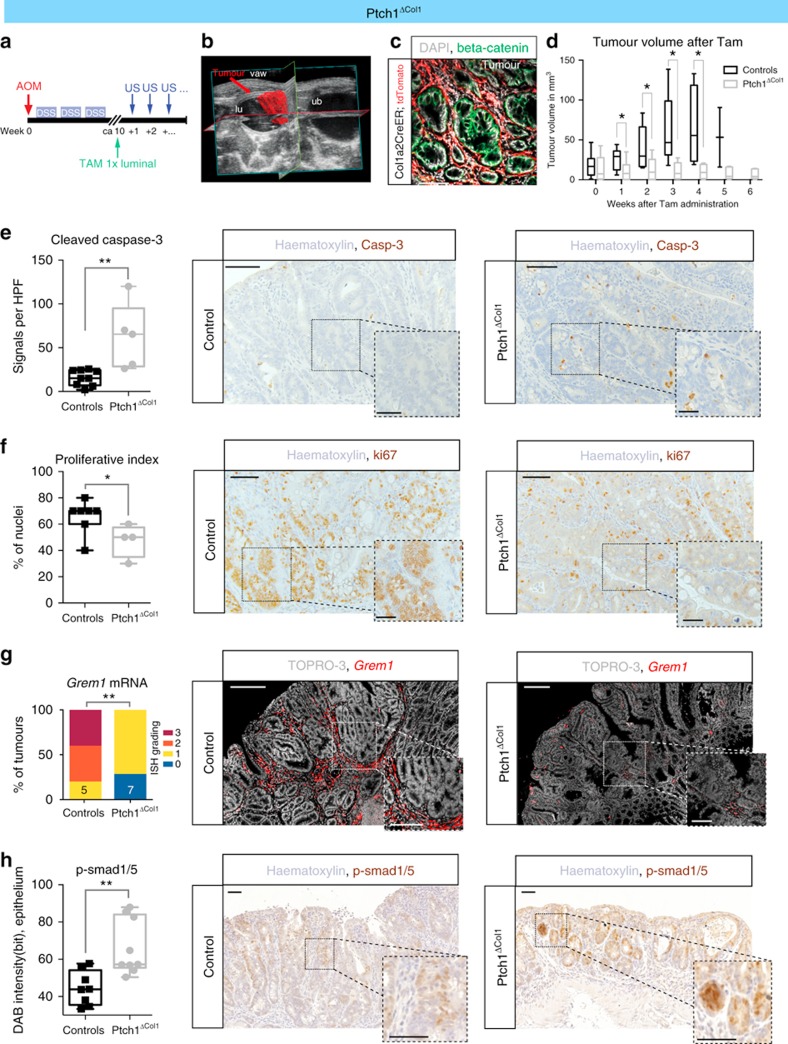# Erratum: Stromal Hedgehog signalling is downregulated in colon cancer and its restoration restrains tumour growth

**DOI:** 10.1038/ncomms12936

**Published:** 2016-09-13

**Authors:** Marco Gerling, Nikè V.J.A. Büller, Leonard M Kirn, Simon Joost, Oliver Frings, Benjamin Englert, Åsa Bergström, Raoul V. Kuiper, Leander Blaas, Mattheus C. B. Wielenga, Sven Almer, Anja A. Kühl, Erik Fredlund, Gijs R. van den Brink, Rune Toftgård

Nature Communications 7:12321 doi:10.1038/ncomms12321 (2016); Published 5 Aug 2016; Updated 13 Sep 2016

In Fig. 6f of this Article, the labelling of the two immunohistochemistry images was inadvertently changed from ‘Haematoxylin, ki67' to ‘Haematoxylin, Casp-3' during the production process. The correct version of Fig. 6 appears below as Fig. 1.

## Figures and Tables

**Figure 1:**