# Supplementary material for: Stromal Hedgehog signalling is downregulated in colon cancer and its restoration restrains tumour growth
Source: Nat Commun. 2016 Aug 5;7:12321. doi: 10.1038/ncomms12321 (PMC4980446; doi:10.1038/ncomms12321)
Supplement: Supplementary Information — Supplementary Figures 1-9 and Supplementary Tables 1-6. [file ncomms12321-s1.pdf]

Supplementary figure 1

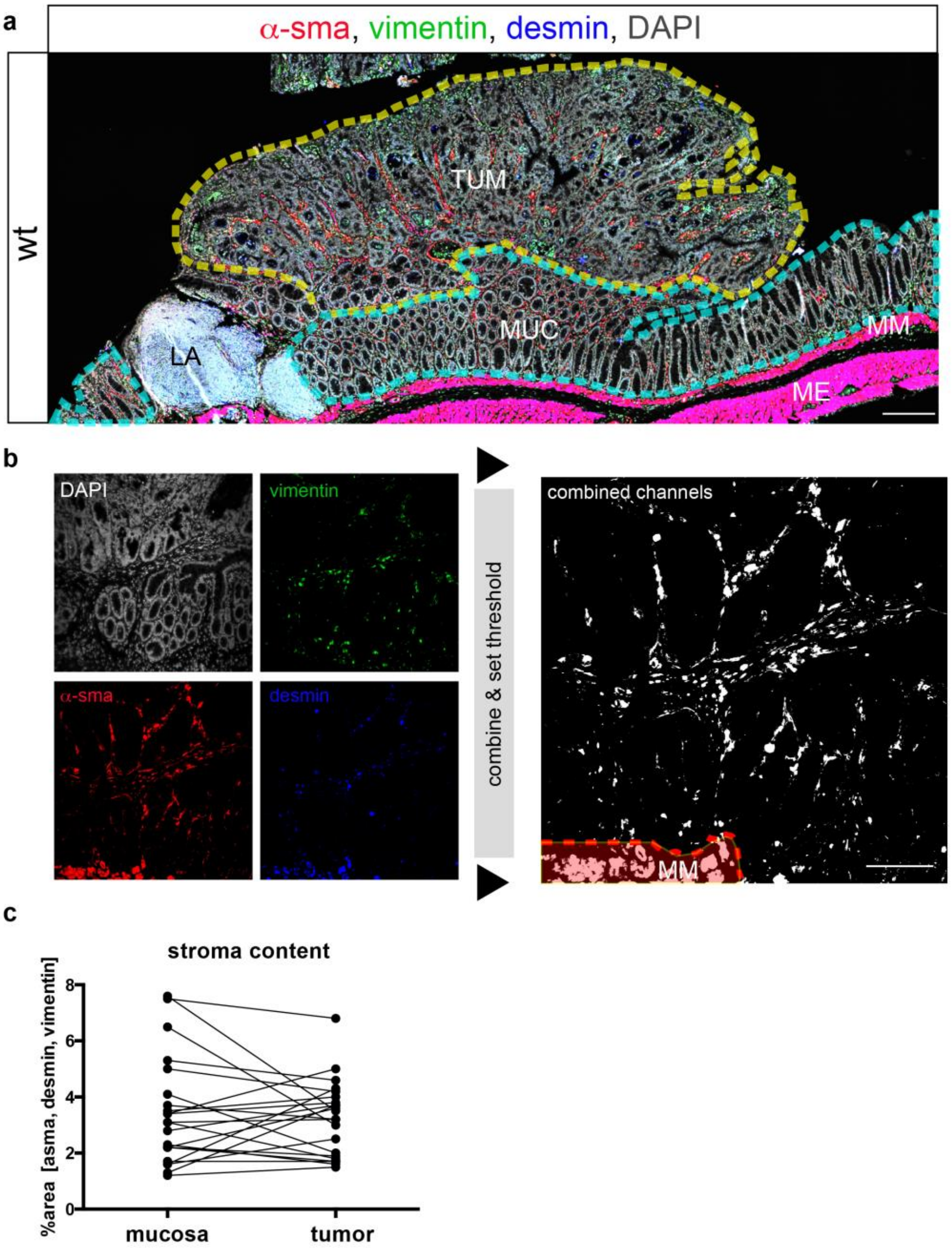

Supplementary Figure 1

Quantification of stromal content in AOM/DSS-induced tumours. a) Representative triple-IF for alpha smooth muscle actin (red), vimentin (green), and desmin (blue) in a tumour from a wt mouse. Nuclear stain: DAPI. Yellow dashed line indicates tumour border, blue indicates normal adjacent mucosa. TUM: tumour, MUC: (non-neoplastic) mucosa; LA: lymphatic aggregate; MM: *muscularis mucosae*; ME: *muscularis externa*. **b)** Example of the quantification process: red, green, and blue channels were combined into a single 8-bit image and the area was then assessed. The *muscularis mucosae* was excluded (red area in right panel). **c)** Quantification for n=23 tumours from n=9 wt mice; adjacent mucosal areas were used in pairwise comparisons to account for staining heterogeneity; p=0.61 (paired t-test).

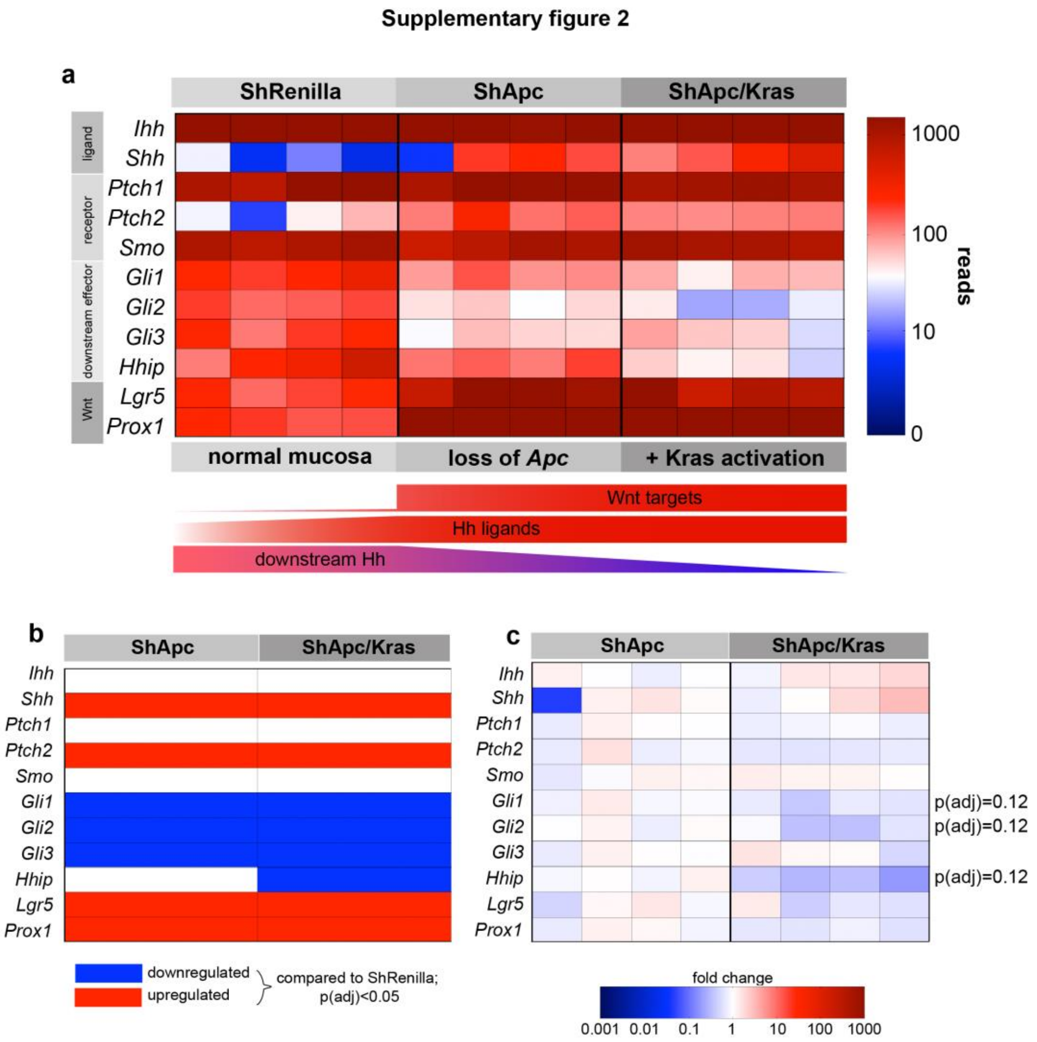

Supplementary Figure 2

Hh expression in an ShRNA model of murine colon tumours

a) Illustration of the absolute RNA reads in ShRenilla controls (normal mucosa, n=4), ShApc-driven tumours (n=4), and ShApc-driven tumours with additional *Kras* mutation (n=4). Each column represents high-throughput RNA sequencing data from one individual mouse. Data are based on GEO dataset GSE67186. **b)** Differential expression of the indicated transcripts in comparison to normal mucosa (ShRenilla controls). Red cell indicates significant upregulation, blue cell indicates significant downregulation; fdr<0.05. **c)** Illustration of expression fold changes in each biological replicate (n=4) of ShApc-driven and ShApc/Kras-driven tumours relative to the average expression over all ShApc replicates. Note that - while not significant - *Gli1*, *Gli2,* and *Hhip* expression is consistently downregulated in all ShApc/Kras samples. The fdr-adjusted p-values for differential expression of *Gli1*, *Gli2,* and *Hhip* are stated on the right.

Supplementary figure 3

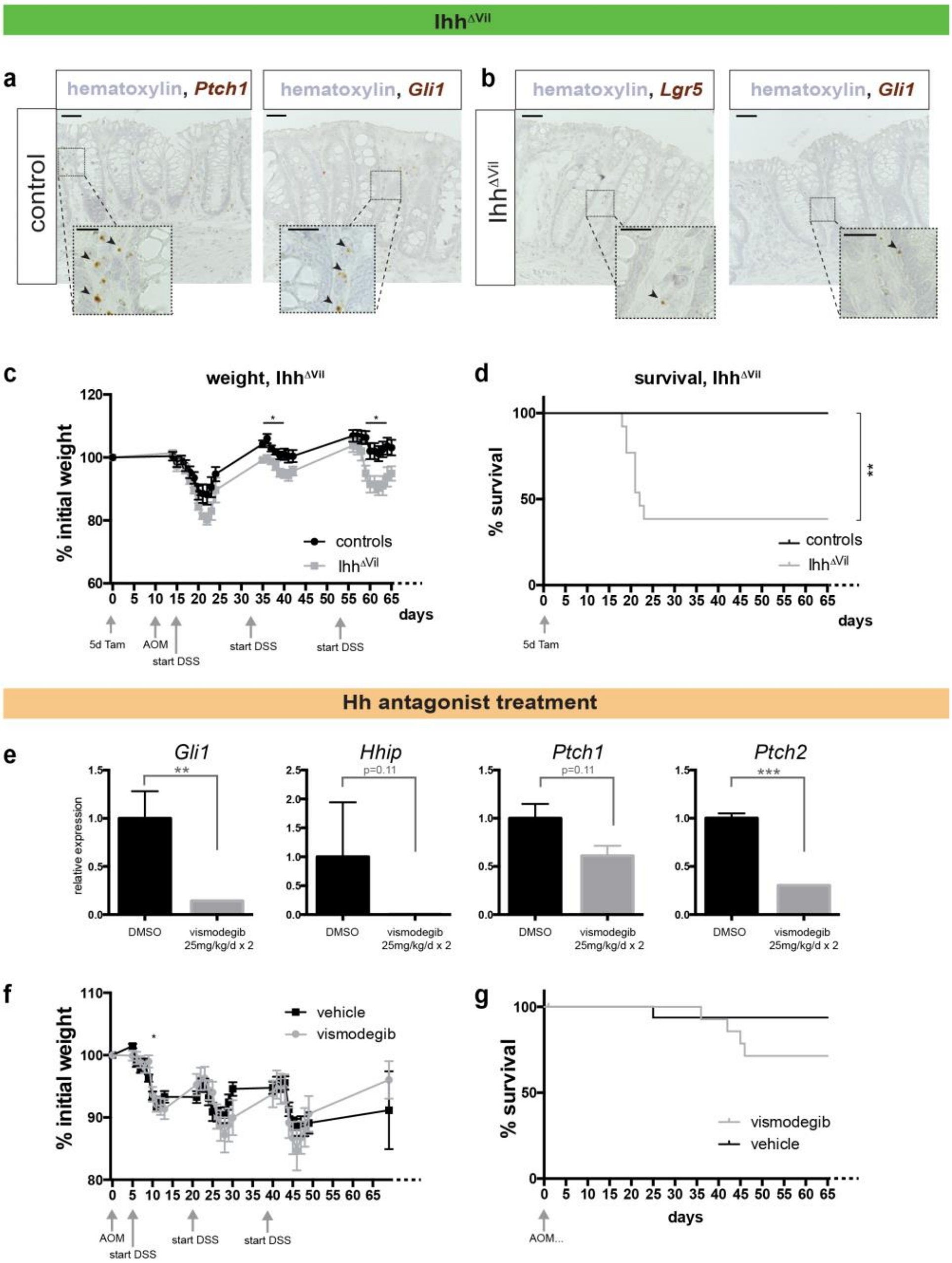

Supplementary Figure 3

Effect of reduced Hh signalling on the course of colitis

RNA ISH for *Ptch1* and *Gli1* in **a)** control mice and **b)** *Ihh*$^{\Delta Vil}$ mice (related to figure 2); representative of n=5 mice/group, scale bars: 20/10µm. **c)** Weight curves of *IhhΔ*^{Vil} mice and controls (related to Fig. 2) during 3 cycles of DSS. Only mice that reached the endpoint are shown (n=5 *IhhΔ*^{Vil} mice and n=11 controls). Weight on days 35–40 and 59–64 was significantly different, $p < 0.05$ (t-test without multiple-test correction). **d)** Survival curves for all mice in this experiment (n=13 *IhhΔ*^{Vil} mice and n=11 controls); the first course of DSS was associated with increased mortality in the *Ihh*$^{\Delta Vil}$ group; log-rank p=0.0019. For the subsequent DSS cycles, the concentration of DSS was lowered from 1.75% to 1.25%, after which no further mortality was observed (described in more detail in *Methods*). **e)** Relative gene expression for the indicated transcripts from wt mice treated with vismodegib, 25 mg/kg bodyweight twice daily (n=3) or vehicle (DMSO, n=3), p=0.0096 (*Gli1*), p=0.11 (*Hhip*), p=0.11 (*Ptch1*), p<0.0001 (*Ptch2*, all t-

tests, fdr-adjusted). **f)** Weight curves during AOM/DSS treatment of mice treated with vismodegib or vehicle (related to figure 2f). Weight was different only on day 9 (p=0.023; t-test without multiple-test correction). **g)** Survival curves, related to (f). Differences were not significant (log-rank p=0.12)

Supplementary figure 4

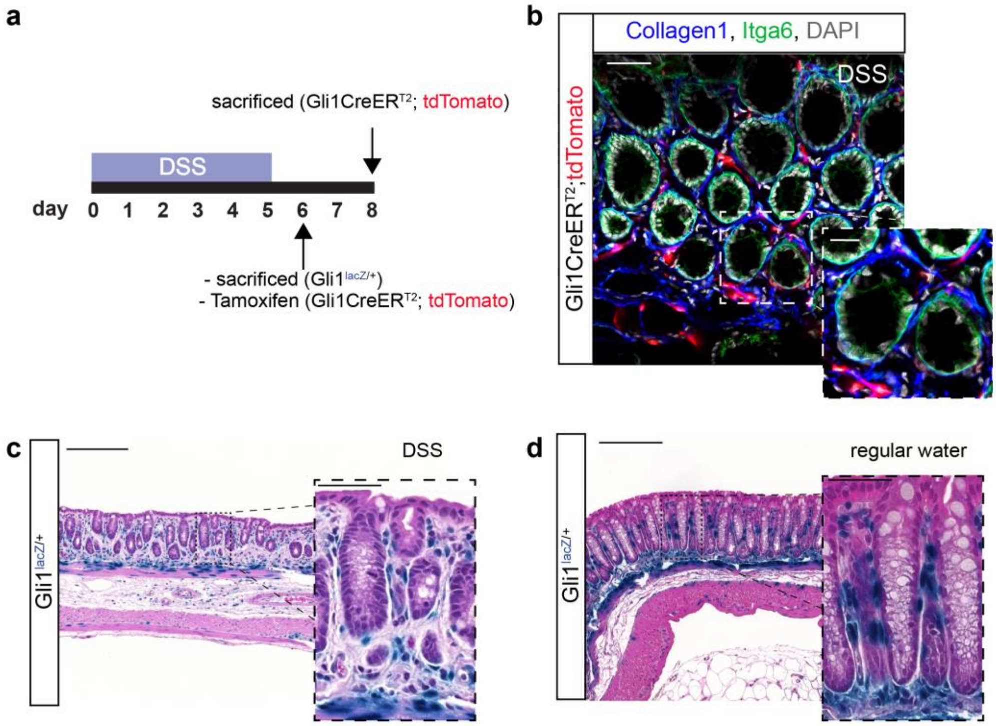

Supplementary Figure 4

Gli1-expressing cells in DSS colitis

a) Schematic of the protocol: 3% DSS was given for 5 days, *Gli1$^{lacZ/+}$* mice (n=3) were sacrificed on day 6 (stage of active colitis), *Gli1CreER^{T2};R26-LSL-tdTomato* mice (n=3) were injected with 5 mg Tam i.p. on day 6 and sacrificed 72h later. **b)** Confocal image of tdTomato fluorescence and staining with antibodies against Collagen 1 (blue, stroma) and Itα6 (green, epithelium). Scale bars 100/20µm; representative of n=3 mice; epithelial tdTomato fluorescence was never observed. **c)** Representative histology (H&E and X-gal staining) of a *Gli1$^{lacZ/+}$* mouse with acute colitis and **d)** a littermate *Gli1$^{lacZ/+}$* mouse that received regular drinking water. Epithelial X-gal staining was never observed. Scale bars 200/50µm; representative of n=3 mice.

Supplementary figure 5

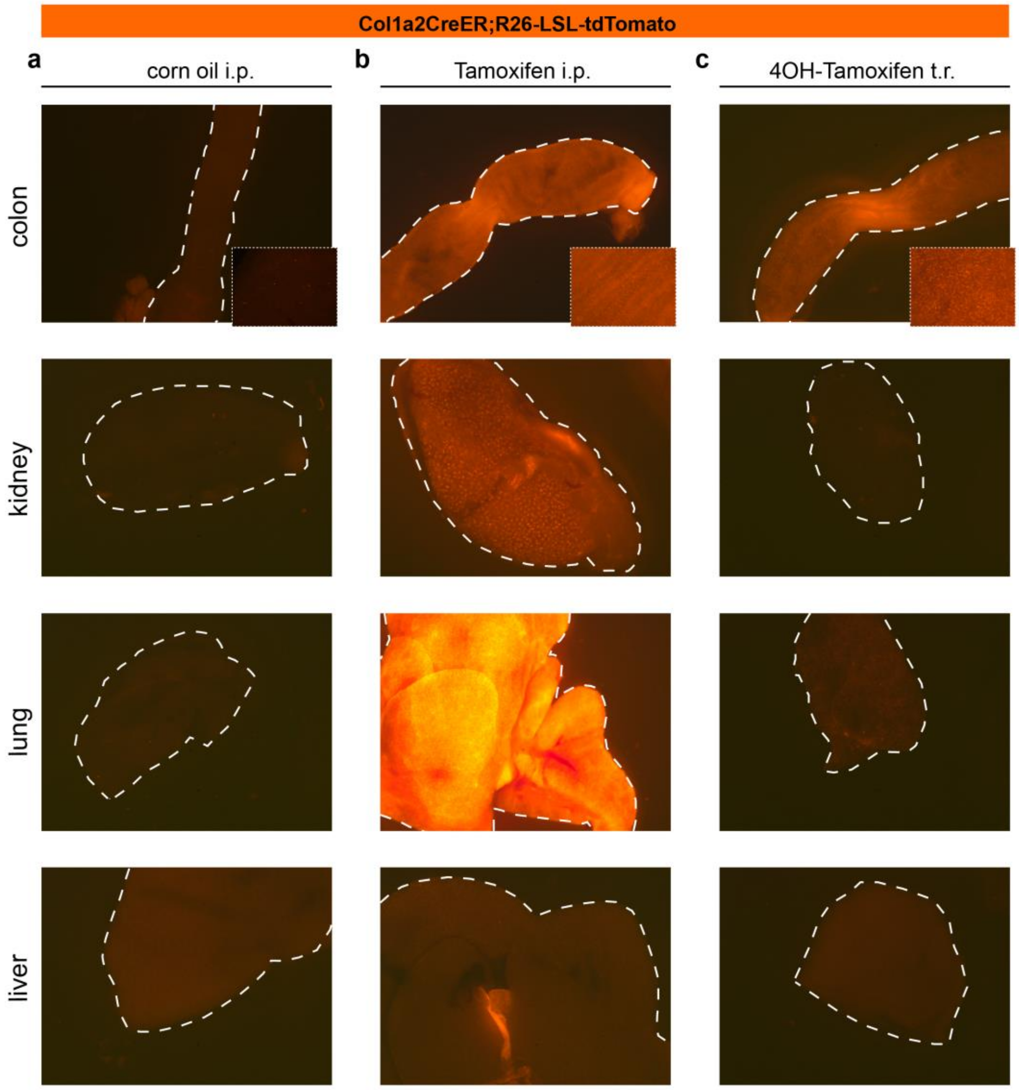

Supplementary Figure 5

Differences between localised and systemic activation of *Col1a2CreER*

Comparison of Cre-mediated recombination between inducible *Col1a2CreER;R26-LSL-tdTomato* reporter mice injected with corn oil **(a)**, injected intraperitoneally (i.p.) with tamoxifen **(b)** or given 4OH-tamoxifen transrectally (t.r.) **(c)** at 10 weeks of age (related to **Figure 3e**). The systemic administration of 5 mg Tam led to recombination in many organs, including colon, kidney, lung, and liver. In the i.p.-treated animals, Tomato^{+} cells were associated with kidney glomeruli, widely distributed in the lung, and located predominantly around the major bile ducts in the liver, while upon t.r. administration, recombination is most frequent in the colon; note that i.p. administration is associated with higher recombination frequency in the *muscularis externa* (oblique longitudinal cells in b, magnified insets). Animals were sacrificed 7 days after Tam treatment. Images representative of n=7 i.p.-treated animals, n=7 i.r.-treated mice, and n=4 oil-treated controls. Images were acquired with a Zeiss SteREO Discovery.V20 microscope with a 0.63X lens at minimum zoom (resulting in 4.7x magnification), taken with an AxioCam MR3 camera and Zeiss Zen 2012 software v 1.1.1.0.

Supplementary figure 6

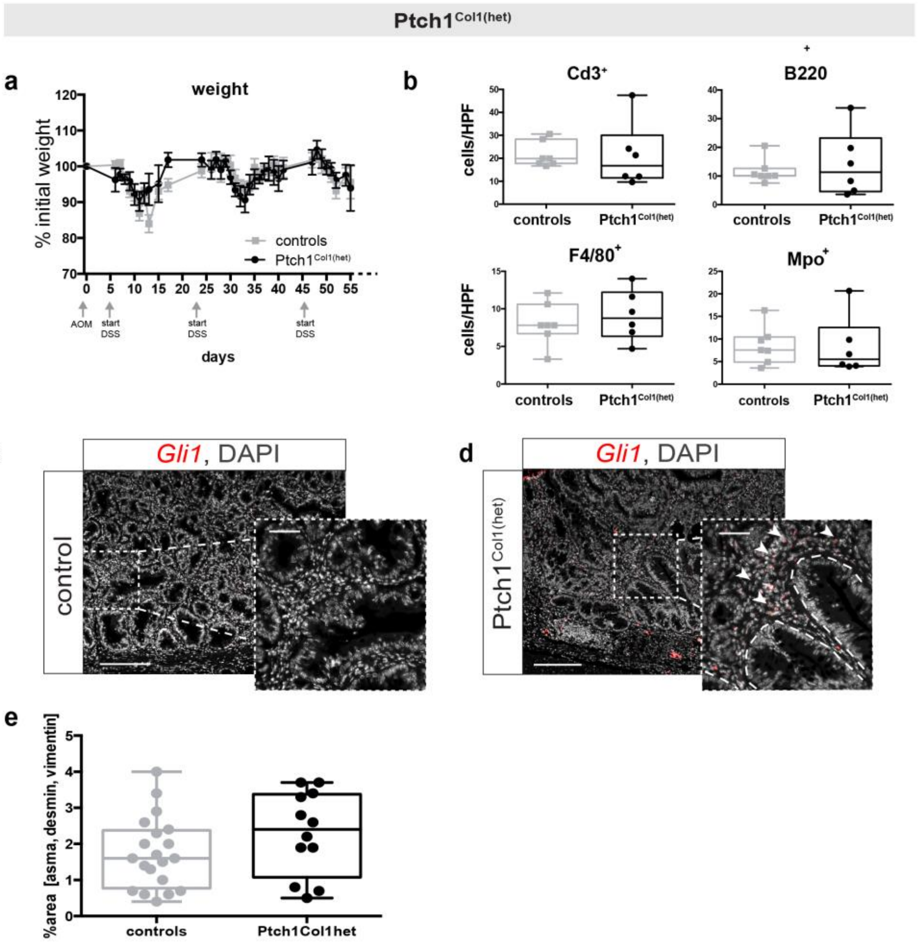

Supplementary Figure 6

***Ptch1*$^{Col(het)}$ mice after treatment with AOM/DSS**

a) Weight curves of n=12 *Ptch1*$^{Col1(het)}$ mice compared to n=12 littermate controls (lacking the *Cre* allele, the floxed *Ptch1* allele or both); day 0: AOM injection (related to **Fig. 4**). Weight was significantly different on day 17 only (p=0.022; t-tests without correction for multiple testing). Survival was not significantly different (not shown). **b)** Immune cell abundance in non-malignant mucosa of the indicated genotypes at the endpoint of the AOM/DSS protocol; p=0.805 (Cd3); p=0.915 (B220); p=0.805 (Mpo); p=0.805 (F4/80; all t-tests; adjusted p-values [fdr] are presented). Box-and-whisker plots, whiskers represent minima and maxima. IHC for a subset of n=6 *Ptch1*$^{Col1(het)}$ mice and n=7 controls (as defined in a). **c** and **d):** Fluorescence RNA ISH for *Gli1* mRNA in **c)** control mice (related to **Fig, 4b**) and **d)** *Ptch1*$^{Col1(het)}$ mice. *Gli1* expression was observed in the stroma of *Ptch1*$^{Col1(het)}$ mice (arrowheads, dashed line indicates epithelial border); scale bars: 200/50 µm. **e)** Quantification of stromal content with triple-IF (as described in Supplementary Fig. 1), for n=20 tumours from n=6 littermate controls and n=12 tumours from n=6 *Ptch1*$^{Col1(het)}$ mice; p=0.158, t-test.

Supplementary figure 7

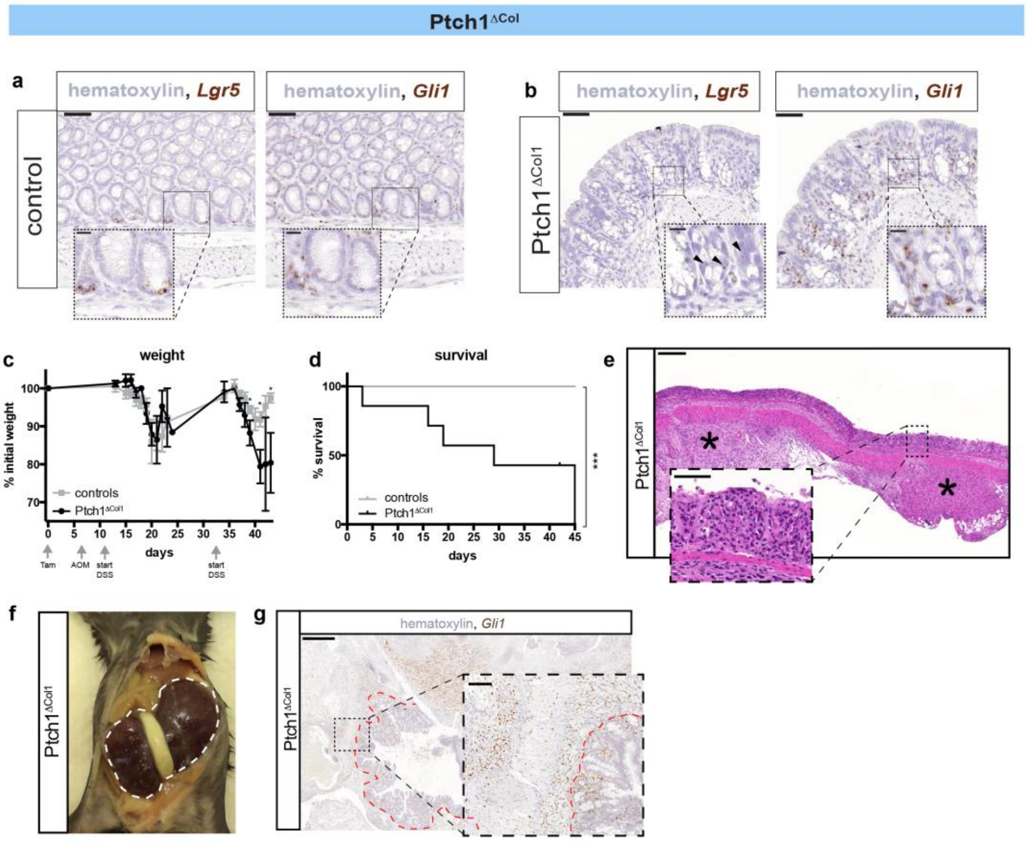

Supplementary Figure 7

Homozygously floxed *Ptch1*$^{\Delta Col1}$ mice

a) Representative ISH images showing *Lgr5* and *Gli1* expression in **a)** a control mouse (lacking *Col1a2CreER* allele) and **b)** a *Ptch1*$^{\Delta Col1}$ mouse 7d after 5 mg Tam i.p. (related to Figure 5). Arrows in left panel magnification in (b) show dots indicating *Lgr5* expression. Scale bars 50/20µm. **c)** Relative weight of mice during two cycles of DSS; n=7 *Ptch1*$^{\Delta Col1}$ mice and n=9 controls (lacking Cre recombinase). Differences were significant on days 39 (p=0.037), 41 (p=0.011), and 43 (p=0.004; all t-tests without correction for multiple testing). **d)** Survival curves; log-rank p=0.0002. One *Ptch1*$^{\Delta Col1}$ mouse was found dead on day 3 after Tam treatment (t.r.); one *Ptch1*$^{\Delta Col1}$ mouse was sacrificed due to self-inflicted wounds (day 42, not counted as experiment-related event). The other mice were sacrificed following ethical guidelines. **e)** H&E staining of a colon section from a *Ptch1*$^{\Delta Col1}$ mouse sacrificed on day 16 (1st cycle of DSS-colitis) due to severe weight loss and bloody diarrhoea. Note the almost complete destruction of the epithelial lining (magnification) as well as stromal proliferation (asterisks, related to panels f and g). Scale bars: 200/50 µm. **f)** Macroscopic appearance of a large, cystic stromal tumour (white dashed line) in a 6 month old female *Ptch1*$^{\Delta Col1}$ mouse, diagnosed 8 days after application of 4OH-Tam t.r. **g)** RNA ISH and hematoxylin staining of the tumour in (d) showing high *Gli1* expression in the stromal tumour.

Supplementary figure 8

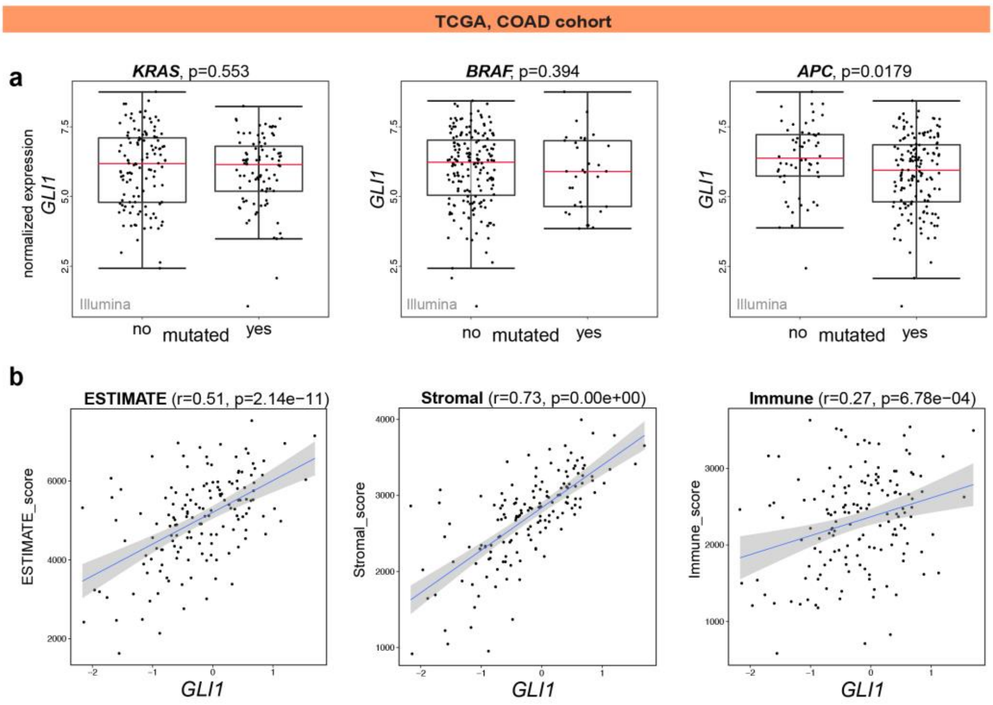

Supplementary Figure 8

Correlation of *GLI1* expression with driver mutations and stromal gene signatures

a) Correlation of *GLI1* mRNA levels with the presence of *KRAS*, *BRAF*, and *APC* mutations; a weak correlation was observed between *APC* mutations and low *GLI1* expression (p-values indicated above the panels). NB: For this analysis, gene expression data were extracted from the TCGA COAD Illumina RNA-seq dataset and correlated to the Illumina mutation data set, as the mutation data corresponding to the Agilent data used for all other TCGA analysis was limited to 53 samples. **b)** Correlation of *GLI1* to stromal gene expression signatures as defined by the ESTIMATE algorithm. A strong positive correlation was observed for *GLI1* and tumour stroma content ("ESTIMATE", left panel, score combines "stromal" and "immune" cell markers). This robust association appeared to be dominated by a very strong correlation between *GLI1* and non-immune cell markers (middle panel, "stromal" gene expression), while genes thought to be expressed predominantly in immune cells contributed to a lesser extent (right panel, "immune" gene expression). Correlation coefficients and p-values are indicated above the panels.

Supplementary figure 9

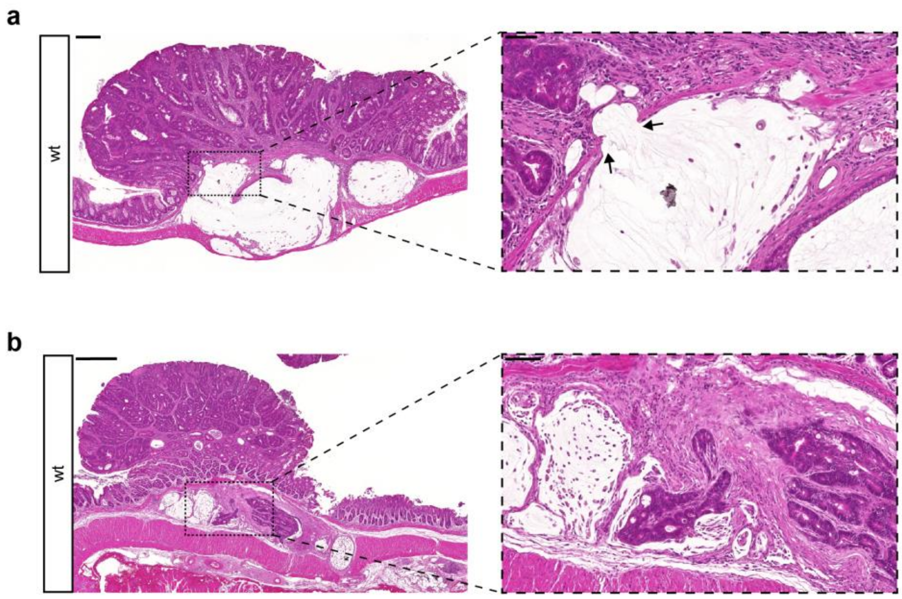

Supplementary Figure 9

Invasive tumours in mice treated with AOM/DSS

Representative examples of invasive tumours from wt C57BL/6 mice at the endpoint of the AOM/DSS protocol. In **a)**, the *muscularis mucosae* appears discontinuous (arrows in magnified image, right panel), while in **b)**, malignant cells are found below the *muscularis mucosae,* together with desmoplastic stroma and mucus lakes, strongly indicating invasiveness. Scale bars 200/50 μm (a), 500/100 μm (b).

Supplementary Table 1 related to Figure 5a **GSEA: 50 top and bottom genes of rank-ordered gene list using GSEA (*Col1a2CreER; Ptch1fl/fl* mice vs. controls).** Note that the floxed sites in the *Ptch1* construct do not include the start codon, thus mRNA is still present, yet the protein is truncated

Rank	GENE	GENE TITLE	GSEA SCORE	fc 'Activated vs Controls'
1	**GLI1**	glioma-associated oncogene homolog 1 (zinc finger protein)	**2.198054**	2.5635
2	**PTCH2**	patched homolog 2 (Drosophila)	**2.054821**	4.5748
3	**CILP**	cartilage intermediate layer protein, nucleotide pyrophosphohydrolase	**2.0368161**	2.4724
4	**CLDN1**	claudin 1	**1.9922407**	2.2774
5	**PRM1**	protamine 1	**1.8895104**	2.4547
6	**HHIP**	hedgehog interacting protein	**1.6809531**	2.2774
7	**PLCD3**	phospholipase C, delta 3	**1.6097883**	2.7429
8	**TMEM100**	transmembrane protein 100	**1.5451641**	2.9679
9	**PDGFC**	platelet derived growth factor C	**1.4554031**	1.8664
10	**MFAP5**	microfibrillar associated protein 5	**1.4167516**	1.8953
11	**PTCH1**	patched homolog 1 (Drosophila)	**1.3934745**	1.8568
12	**GPC3**	glypican 3	**1.382012**	4.1695
13	**PENK**	proenkephalin	**1.2920026**	1.6923
14	**KRT23**	keratin 23 (histone deacetylase inducible)	**1.290661**	1.6043
15	**MEST**	mesoderm specific transcript homolog (mouse)	**1.2704059**	2.7103
16	**BDKRB1**	bradykinin receptor B1	**1.2679647**	1.6701
17	**TBX2**	T-box 2	**1.2547828**	1.8002
18	**S100B**	S100 calcium binding protein B	**1.1521394**	1.4842
19	**RBP4**	retinol binding protein 4, plasma	**1.1485647**	1.6634
20	**HIST1H1B**	histone cluster 1, H1b	**1.1437567**	1.7228
21	**SERPINF1**	serpin peptidase inhibitor, clade F (alpha-2 antiplasmin, pigment epithelium derived factor), member 1	**1.1431973**	1.5392
22	**PCSK5**	proprotein convertase subtilisin/kexin type 5	**1.1261085**	1.8063
23	**VIT**	vitrin	**1.1238635**	1.6657
24	**KCNE4**	potassium voltage-gated channel, Isk-related family, member 4	**1.1209518**	1.9292
25	**LAMA2**	laminin, alpha 2 (merosin, congenital muscular dystrophy)	**1.1062077**	1.7147
26	**FOXL1**	forkhead box L1	**1.092439**	2.2535
27	**ITGBL1**	integrin, beta-like 1 (with EGF-like repeat domains)	**1.087607**	2.447
28	**ACTN2**	actinin, alpha 2	**1.07581**	1.6955
29	**LRRC17**	leucine rich repeat containing 17	**1.0697056**	1.6876
30	**PRRX1**	paired related homeobox 1	**1.0679772**	1.5669
31	**GDF10**	growth differentiation factor 10	**1.0605694**	2.2277
32	**ANGPTL7**	angiopoietin-like 7	**1.0541155**	1.4952
33	**GPX3**	glutathione peroxidase 3 (plasma)	**1.0470741**	1.5165
34	**TM6SF2**	transmembrane 6 superfamily member 2	**1.042523**	1.3988
35	**GSTA4**	glutathione S-transferase A4	**1.0261525**	1.4115
36	**HSPB1**	heat shock 27kDa protein 1	**1.0000974**	1.492
37	**SPRR2B**	small proline-rich protein 2B	**0.99962336**	1.5011
38	**MFAP4**	microfibrillar-associated protein 4	**0.99540406**	1.5203
39	**HOXC6**	homeobox C6	**0.9900814**	1.532
40	**NPPC**	natriuretic peptide precursor C	**0.98975426**	1.4496
41	**ABCA6**	ATP-binding cassette, sub-family A (ABC1), member 6	**0.9773975**	1.4201
42	**E2F2**	E2F transcription factor 2	**0.9580215**	1.3635
43	**CCL17**	chemokine (C-C motif) ligand 17	**0.9537584**	1.8185
44	**TGM5**	transglutaminase 5	**0.94943637**	1.3984
45	**TMEM132E**	transmembrane protein 132E	**0.94784576**	1.4223
46	**PPP1R15A**	protein phosphatase 1, regulatory (inhibitor) subunit 15A	**0.94161767**	1.3372
47	**KL**	klotho	**0.9358641**	1.4478
48	**PPBP**	pro-platelet basic protein (chemokine (C-X-C motif) ligand 7)	**0.93048644**	1.7411
49	**TSPAN11**	tetraspanin 11	**0.92680705**	1.7646
50	**TCF19**	transcription factor 19 (SC1)	**0.92629445**	1.5028

suppl. table 1 ctd.				
-50	**RGS13**	regulator of G-protein signalling 13	**-0.8367354**	-1.3837
-49	**C1QTNF3**	C1q and tumor necrosis factor related protein 3	**-0.84039265**	-1.5754
-48	**PAX5**	paired box gene 5 (B-cell lineage specific activator)	**-0.84173954**	-1.5278
-47	**SNORA21**	small nucleolar RNA, H/ACA box 21	**-0.84259486**	-1.2937
-46	**CNTN6**	contactin 6	**-0.84389216**	-1.3936
-45	**FAIM3**	Fas apoptotic inhibitory molecule 3	**-0.8490927**	-2.2154
-44	**SLC16A12**	solute carrier family 16, member 12 (monocarboxylic acid transporter 12)	**-0.84925187**	-1.3605
-43	**GP2**	glycoprotein 2 (zymogen granule membrane)	**-0.8502086**	-1.3464
-42	**CXCL12**	chemokine (C-X-C motif) ligand 12 (stromal cell-derived factor 1)	**-0.85306937**	-1.3454
-41	**WNT2B**	wingless-type MMTV integration site family, member 2B	**-0.8552463**	-1.3915
-40	**CCR6**	chemokine (C-C motif) receptor 6	**-0.85580623**	-1.3966
-39	**CRISP3**	cysteine-rich secretory protein 3	**-0.86031723**	-1.3212
-38	**RGS8**	regulator of G-protein signalling 8	**-0.8608562**	-1.3618
-37	**SLC6A12**	solute carrier family 6 (neurotransmitter transporter, betaine/GABA), member 12	**-0.86216533**	-1.3284
-36	**IL21R**	interleukin 21 receptor	**-0.86512524**	-1.4653
-35	**MYCBPAP**	MYCBP associated protein	**-0.8686706**	-1.3323
-34	**GPR174**	G protein-coupled receptor 174	**-0.87022036**	-1.4919
-33	**TMEM35**	transmembrane protein 35	**-0.87512726**	-1.31
-32	**TREML2**	triggering receptor expressed on myeloid cells-like 2	**-0.8796631**	-1.3884
-31	**ID4**	inhibitor of DNA binding 4, dominant negative helix-loop-helix protein	**-0.88256747**	-1.3727
-30	**MME**	membrane metallo-endopeptidase (neutral endopeptidase, enkephalinase)	**-0.8843302**	-1.3901
-29	**HAAO**	3-hydroxyanthranilate 3,4-dioxygenase	**-0.88462263**	-1.4897
-28	**SNORA31**	small nucleolar RNA, H/ACA box 31	**-0.8923063**	-1.7994
-27	**SLC5A6**	solute carrier family 5 (sodium-dependent vitamin transporter), member 6	**-0.8936503**	-1.4336
-26	**PCDH15**	protocadherin 15	**-0.89943933**	-1.381
-25	**LGR5**	leucine-rich repeat-containing G protein-coupled receptor 5	**-0.9052235**	-1.3681
-24	**SERPINB9**	serpin peptidase inhibitor, clade B (ovalbumin), member 9	**-0.9127192**	-1.32
-23	**COLEC10**	collectin sub-family member 10 (C-type lectin)	**-0.91364694**	-1.4873
-22	**ADRA2B**	adrenergic, alpha-2B-, receptor	**-0.9249009**	-1.4726
-21	**HVCN1**	hydrogen voltage-gated channel 1	**-0.92571616**	-1.3393
-20	**H2AFB1**	H2A histone family, member B1	**-0.93038845**	-1.4408
-19	**GPT2**	glutamic pyruvate transaminase (alanine aminotransferase) 2	**-0.94319946**	-1.4329
-18	**GABRP**	gamma-aminobutyric acid (GABA) A receptor, pi	**-0.94663846**	-1.4261
-17	**SPIB**	Spi-B transcription factor (Spi-1/PU.1 related)	**-0.958244**	-1.5758
-16	**CDC14B**	CDC14 cell division cycle 14 homolog B (S. cerevisiae)	**-0.97026914**	-1.422
-15	**BMP6**	bone morphogenetic protein 6	**-0.9768685**	-1.4309
-14	**CXCL10**	chemokine (C-X-C motif) ligand 10	**-0.98531926**	-1.4643
-13	**SEC14L4**	SEC14-like 4 (S. cerevisiae)	**-0.9917472**	-1.638
-12	**EDIL3**	EGF-like repeats and discoidin I-like domains 3	**-1.0087286**	-1.4263
-11	**FGF11**	fibroblast growth factor 11	**-1.0113168**	-1.5591
-10	**SLC7A11**	solute carrier family 7, (cationic amino acid transporter, y+ system) member 11	**-1.0566427**	-1.5637
-9	**PGLYRP2**	peptidoglycan recognition protein 2	**-1.0835605**	-1.7096
-8	**KRTAP16-1**	keratin associated protein 16-1	**-1.0859587**	-1.6416
-7	**SNORA68**	small nucleolar RNA, H/ACA box 68	**-1.0964037**	-1.5143
-6	**IFIT3**	interferon-induced protein with tetratricopeptide repeats 3	**-1.1234238**	-1.4996
-5	**FCRL1**	Fc receptor-like 1	**-1.1421783**	-1.7001
-4	**AZGP1**	alpha-2-glycoprotein 1, zinc	**-1.15433**	-1.6202
-3	**KCNN3**	potassium intermediate/small conductance calcium-activated channel, subfamily N, member 3	**-1.2254028**	-1.656
-2	**ADCY2**	adenylate cyclase 2 (brain)	**-1.2582403**	-1.7341
-1	**UNC5A**	unc-5 homolog A (C. elegans)	**-1.5228664**	-2.0652

Supplementary Table 2 — **GSEA, hedgehog signaling**

related to Figure 5b

Gene set derived from MSigD, M211, Schaefer et al., Nucleic Acids Res. 2009 Jan (37), PMID 18832364

GENE_SYMBOL	GENE_TITLE	RANK METRIC SCORE	RUNNING ES	CORE ENRICHMENT
**PTCH2**	patched homolog 2 (Drosophila)	2.054821014	0.20855872	**Yes**
**HHIP**	hedgehog interacting protein	1.680953145	0.37899724	**Yes**
**PTCH1**	patched homolog 1 (Drosophila)	1.39347446	0.52016675	**Yes**
**TGFB2**	transforming growth factor, beta 2	0.852665484	0.60172987	**Yes**
**STIL**	SCL/TAL1 interrupting locus	0.767577231	0.6763245	**Yes**
**GLI2**	GLI-Kruppel family member GLI2	0.610204458	0.72594833	**Yes**
**LRP2**	low density lipoprotein-related protein 2	0.20916611	0.5701352	No
**HHAT**	hedgehog acyltransferase	0.157404467	0.5300181	No
**ARRB2**	arrestin, beta 2	0.134484276	0.51415133	No
**BOC**	Boc homolog (mouse)	-2.22E-04	0.3100679	No
**PTHLH**	parathyroid hormone-like hormone	-0.010101718	0.2966908	No
**DHH**	desert hedgehog homolog (Drosophila)	-0.029842019	0.26637954	No
**CDON**	Cdon homolog (mouse)	-0.031355239	0.26645985	No
**SMO**	smoothened homolog (Drosophila)	-0.089872085	0.18184754	No
**AKT1**	v-akt murine thymoma viral oncogene homolog 1	-0.090706363	0.18946603	No
**SHH**	sonic hedgehog homolog (Drosophila)	-0.104446813	0.17842714	No
**PIK3CA**	phosphoinositide-3-kinase, catalytic, alpha polypeptide	-0.133653909	0.14847113	No
**LRPAP1**	low density lipoprotein receptor-related protein associated protein 1	-0.14296332	0.15065317	No
**ADRBK1**	adrenergic, beta, receptor kinase 1	-0.155708417	0.14967316	No
**PIK3R1**	phosphoinositide-3-kinase, regulatory subunit 1 (p85 alpha)	-0.175553352	0.14028402	No
**GAS1**	growth arrest-specific 1	-0.411558896	-0.002220137	No
**IHH**	Indian hedgehog homolog (Drosophila)	-0.611989498	0.016471697	No

**Supplementary Table 3**<br><br>related to Figure 5b	**GSEA, brush border**<br><br>Gene set derived from gene ontology term GO:0005903, *"Dense covering of microvilli on the apical surface of epithelial cells in tissues such as the intestine, kidney, and choroid plexus; the microvilli aid absorption by increasing the surface area of the cell."*

**GENE_SYMBOL**	**GENE_TITLE**	RANK METRIC SCORE	RUNNING ES	CORE ENRICHMENT
**ALPI**	alkaline phosphatase, intestinal	0.567796648	0.23066677	**Yes**
**CLIC1**	chloride intracellular channel 1	0.313766301	0.2857041	**Yes**
**MYO1A**	myosin IA	0.286958218	0.40108258	**Yes**
**ITPR3**	inositol 1,4,5-triphosphate receptor, type 3	0.275004119	0.5187957	**Yes**
**SLC22A12**	solute carrier family 22 (organic anion/cation transporter), member 12	0.196153551	0.53677195	**Yes**
**ATP6V0A4**	ATPase, H+ transporting, lysosomal V0 subunit a4	0.173612088	0.5912813	**Yes**
**B4GALT1**	UDP-Gal:betaGlcNAc beta 1,4- galactosyltransferase, polypeptide 1	0.172640741	0.6692742	**Yes**
**ESPN**	espin	0.11148902	0.6440812	No
**VIL1**	villin 1	0.093789756	0.6644674	No

**Supplementary Table 4**	**GSEA, *Lgr5* related**			
related to Figure 5b	Gene set derived from Dalerba *et al.*, Nature Biotechnology, (12) 2011, PMID 22081019			
**GENE_SYMBOL**	**GENE_TITLE**	RANK METRIC SCORE	RUNNING ES	CORE ENRICHMENT
**BMI1**	B lymphoma Mo-MLV insertion region (mouse)	0.765854597	0.09156098	No
**OLFM4**	olfactomedin 4	0.244901061	-0.030712042	No
**PTPRO**	protein tyrosine phosphatase, receptor type, O	0.081057861	-0.21209067	No
**AXIN2**	axin 2 (conductin, axil)	0.079184368	-0.20466925	No
**EGFR**	epidermal growth factor receptor (erythroblastic leukemia viral (v-erb-b) oncogene homolog, avian)	0.025412334	-0.28672633	No
**EZH2**	enhancer of zeste homolog 2 (Drosophila)	0.015512916	-0.30179358	No
**TSPAN6**	tetraspanin 6	-0.039914809	-0.38854417	No
**MLLT10**	myeloid/lymphoid or mixed-lineage leukemia (trithorax homolog, Drosophila); translocated to, 10	-0.060935132	-0.41584736	No
**GPSM2**	G-protein signalling modulator 2 (AGS3-like, C. elegans)	-0.123392902	-0.49707764	No
**HES1**	hairy and enhancer of split 1, (Drosophila)	-0.130449846	-0.48918146	No
**CXCL2**	chemokine (C-X-C motif) ligand 2	-0.157066375	-0.5041212	No
**AQP1**	aquaporin 1 (Colton blood group)	-0.166807607	-0.49501058	No
**MYC**	v-myc myelocytomatosis viral oncogene homolog (avian)	-0.187595636	-0.49645057	No
**STMN1**	stathmin 1/oncoprotein 18	-0.253885895	-0.53387994	**Yes**
**ASCL2**	achaete-scute complex-like 2 (Drosophila)	-0.26303032	-0.50777704	**Yes**
**DNMT3A**	DNA (cytosine-5-)-methyltransferase 3 alpha	-0.280733645	-0.48522902	**Yes**
**LRIG1**	leucine-rich repeats and immunoglobulin-like domains 1	-0.284747571	-0.45076886	**Yes**
**NOTCH1**	Notch homolog 1, translocation-associated (Drosophila)	-0.304963261	-0.42765117	**Yes**
**RNF43**	ring finger protein 43	-0.316223234	-0.39317778	**Yes**
**DPP4**	dipeptidyl-peptidase 4 (CD26, adenosine deaminase complexing protein2)	-0.338856488	-0.3627233	**Yes**
**CDCA7**	cell division cycle associated 7	-0.375400156	-0.33278096	**Yes**
**KIF12**	kinesin family member 12	-0.431301415	-0.2972948	**Yes**
**RGMB**	RGM domain family, member B	-0.472093225	-0.24997981	**Yes**
**CDK6**	cyclin-dependent kinase 6	-0.590079188	-0.19147776	**Yes**
**CFTR**	cystic fibrosis transmembrane conductance regulator (ATP-binding cassette sub-family C, member 7)	-0.685600936	-0.10954883	**Yes**
**LGR5**	leucine-rich repeat-containing G protein-coupled receptor 5	-0.905223489	0.001910388	**Yes**

**Supplementary Table 5**	**GSEA, *BMP inhibitors***			
related to Figure 5b	Gene set derived from GO term 0030514, "negative regulation of BMP signaling pathway", filter settings as described in Materials & Methods			
**GENE_SYMBOL**	**GENE_TITLE**	RANK METRIC SCORE	RUNNING ES	CORE ENRICHMENT
**CTDSP1**	CTD (carboxy-terminal domain, RNA polymerase II, polypeptide A) small phosphatase 1	0.09614034	-0.30160755	No
**GSK3B**	glycogen synthase kinase 3 beta	0.060752008	-0.3382314	No
**CHRD**	chordin	0.059891008	-0.32064706	No
**SOSTDC1**	sclerostin domain containing 1	0.017844023	-0.38265622	No
**CTDSPL**	CTD (carboxy-terminal domain, RNA polymerase II, polypeptide A) small phosphatase-like	0.012922896	-0.38564318	No
**MAPK1**	mitogen-activated protein kinase 1	-0.03782884	-0.45758638	No
**SMURF1**	SMAD specific E3 ubiquitin protein ligase 1	-0.102476731	-0.52727246	**Yes**
**SMURF2**	SMAD specific E3 ubiquitin protein ligase 2	-0.106276728	-0.49877033	**Yes**
**TMPRSS6**	transmembrane protease, serine 6	-0.11507488	-0.4768934	**Yes**
**MAP3K7**	mitogen-activated protein kinase kinase kinase 7	-0.162428096	-0.4892843	**Yes**
**SKI**	v-ski sarcoma viral oncogene homolog (avian)	-0.207304314	-0.47692716	**Yes**
**BAMBI**	BMP and activin membrane-bound inhibitor homolog (Xenopus laevis)	-0.208975464	-0.4114534	**Yes**
**HFE2**	hemochromatosis type 2 (juvenile)	-0.237576619	-0.36506334	**Yes**
**CER1**	cerberus 1, cysteine knot superfamily, homolog (Xenopus laevis)	-0.282663107	-0.3139815	**Yes**
**NOG**	noggin	-0.299755633	-0.23184884	**Yes**
**CHRDL1**	chordin-like 1	-0.397747695	-0.15753473	**Yes**
**GREM1**	gremlin 1, cysteine knot superfamily, homolog (Xenopus laevis)	-0.69200325	0.009226845	**Yes**

Supplementary Table 6

related to Figure 5c-f

human colon fibroblasts (GSE29316) and murine intestinal mesenchyme (GSE17840) treated with Hh ligand red: significantly upregulated upon Hh ligand treatment, blue: significantly downregulated

GSE29316 – Chen *et al.*			GSE17840 – Zacharias *et al.*				
	Untreated vs. SHH treated			Vehicle vs. Shh		Vehicle vs. Ihh	
Gene	Fold change	P-value (BH-	Gene	Fold change	P-value (BH-	Fold change	P-value (BH-
BAMBI	0.17197006	0.05354276	***Bambi***	0.17201500	0.07754563	0.09012142	0.31758510
BMP4	0.23971321	0.00765592	***Bmp4***	-0.26967559	0.07306182	-0.15313203	0.30216208
BMP5	-0.02885923	0.71015960	***Bmp5***	-0.15226793	0.30025121	-0.27580747	0.15734066
CER1	0.11066081	0.22702320	***Cer1***	-0.15044605	0.07754563	-0.07758434	0.31758510
CHRDL1	0.41957609	0.00034994	***Chrdl1***	-0.16446043	0.26057328	-0.16208432	0.30216208
FOXL1	-1.23472503	0.00000049	***Foxl1***	-0.29174262	0.03091778	-0.20046922	0.19530849
GDF10	0.17679518	0.15139372	***Gdf10***	-0.60403320	0.03091778	-0.37953272	0.20704949
GLI1	-0.55866688	0.00063000	***Gli1***	-0.79410970	0.02004430	-0.98245485	0.00798679
GLI2	-0.21144063	0.06352696	***Gli2***	-0.27549293	0.02937271	-0.37676960	0.01131978
GLI3	1.23973303	0.00000050	***Gli3***	-0.07878173	0.26057328	-0.09462075	0.22536717
GPX3	0.13928383	0.08174368	***Gpx3***	-0.47497599	0.02004430	-0.27905292	0.13538626
GREM1	1.17980510	0.00000015	***Grem1***	0.25820631	0.02004430	0.40374030	0.00474189
HFE2	-0.13517016	0.13938619	***Hfe2***	0.13665878	0.09654544	0.12717822	0.20589278
HHIP	0.05015396	0.58110825	***Hhip***	-0.07450749	0.37373281	-0.08376191	0.31758510
MAP3K7	0.06650297	0.38944629	***Map3k7***	-0.09572554	0.37373281	-0.08308812	0.39957787
NOG	1.18680176	0.00000006	***Nog***	0.03334358	0.75587367	-0.13155465	0.30216208
PTCH1	-1.08333744	0.00005091	***Ptch1***	-0.41723761	0.02004430	-0.42565353	0.01627687
PTCH2	-0.13359180	0.18359789	***Ptch2***	-0.32795230	0.02004430	-0.30777202	0.04036567
SKI	0.07545063	0.46177172	***Ski***	0.24780600	0.02004430	0.12877017	0.20704949
SMO	-0.14299272	0.09446659	***Smo***	0.03699820	0.75587367	-0.05888694	0.55363542
SMURF1	0.06461602	0.40694905	***Smurf1***	0.17993062	0.12616071	0.12381775	0.30596290
SMURF2	-0.39567491	0.00022877	***Smurf2***	-0.17897209	0.07114918	-0.07343600	0.36453459
SOSTDC1	-0.03895256	0.65761576	***Sostdc1***	0.81250873	0.02004430	0.25789511	0.31758510
TMPRSS6	-0.00603307	0.93116259	***Tmprss6***	0.00118962	0.98667039	0.12361946	0.20987309
